# Genetic evidence for asymmetric blocking of higher-order chromatin structure by CTCF/cohesin

**DOI:** 10.1007/s13238-019-00656-y

**Published:** 2019-09-26

**Authors:** Yujia Lu, Jia Shou, Zhilian Jia, Yonghu Wu, Jinhuan Li, Ya Guo, Qiang Wu

**Affiliations:** 1 MOE Key Lab of Systems Biomedicine, Center for Comparative Biomedicine, State Key Lab of Oncogenes and Related Genes, Shanghai Cancer Institute, Joint International Research Laboratory of Metabolic & Developmental Sciences, Institute of Systems Biomedicine, Xin Hua Hospital, Shanghai Jiao Tong University, Shanghai, 200240 China; 2 Present Address: Ming Wai Lau Centre for Reparative Medicine, Karolinska Institutet, Hong Kong, China; 3 Present Address: MRC London Institute of Medical Sciences, Imperial College London, London, W12 0NN UK

**Dear Editor,**

Similar to higher-order folding of polypeptide chains into functional proteins, linear DNA molecules are spatially folded in a hierarchical and dynamic manner into three-dimensional (3D) functional chromatin structures in eukaryotic nuclei (Huang and Wu, 2016; Rowley and Corces, 2018). This dynamic folding is closely related to many nuclear processes such as DNA replication and repair, chromosomal translocation, recombination, and segregation, as well as RNA transcription, splicing, and transport. In particular, dynamic long-distance chromatin looping interactions, which result in close spatial contacts between distal enhancers and target promoters, are thought to play a role in controlling precise spatiotemporal as well as cell-type specific gene expression during animal development (Rowley and Corces, 2018). Mammalian genomes contain numerous noncoding regulatory elements that regulate these dynamic long-distance chromatin looping interactions. Specially, one type of genetic elements, known as insulators, plays a delicate role to ensure proper activation of target promoters by distal enhancers (Huang and Wu, 2016). In mammals, the most prominent insulator-binding protein is CCCTC binding factor (CTCF), an architectural protein with 11 zinc-fingers essential for 3D genome organization (Yin et al., 2017; Wu et al., 2019).

CTCF dynamically and directionally binds to hundreds of thousands genomic sites and this binding is pivotal for its multivalent role in many cellular and developmental processes (Guo et al., 2015; Yin et al., 2017). In particular, CTCF, in collaboration with its associated cohesin complex, determines V(D)J recombination of the *Bcr* (B-cell receptor) and *Tcr* (T-cell receptor) gene clusters in the immune system (Jain et al., 2018; Wu et al., 2019) and promoter choice of the protocadherin (*Pcdh*) gene clusters in the nervous system (Guo et al., 2012; Mountoufaris et al., 2018; Canzio et al., 2019; Wu et al., 2019). The clustered *Pcdh* genes have been used as model genes for investigating mechanisms of higher-order chromatin folding (Fig. 1A). In mice, for example, 58 clustered *Pcdh* genes are linearly organized into three closely-linked clusters of the *α*, *β*, and *γ* (Fig. S1A) (Wu et al., 2001). Spatially, these three *Pcdh* clusters are assembled via CTCF/cohesin-mediated chromatin looping into two TAD-like structures known as subTAD or CCD (Guo et al., 2012, 2015). The entire *Pcdh* locus forms a superTAD that could be disrupted by ablating histone lysine methyltransferase gene *Setdb1*, resulting in aberrant overexpression of the clustered *Pcdh* genes (Jiang et al., 2017). The encoded Pcdh proteins are required for neuronal migration, dendrite self-avoidance and tiling, dendritic spine morphogenesis and elaboration, axon extension and tiling, and neuronal circuit assembly and function in the brain (Mountoufaris et al., 2018).

**Figure 1:**
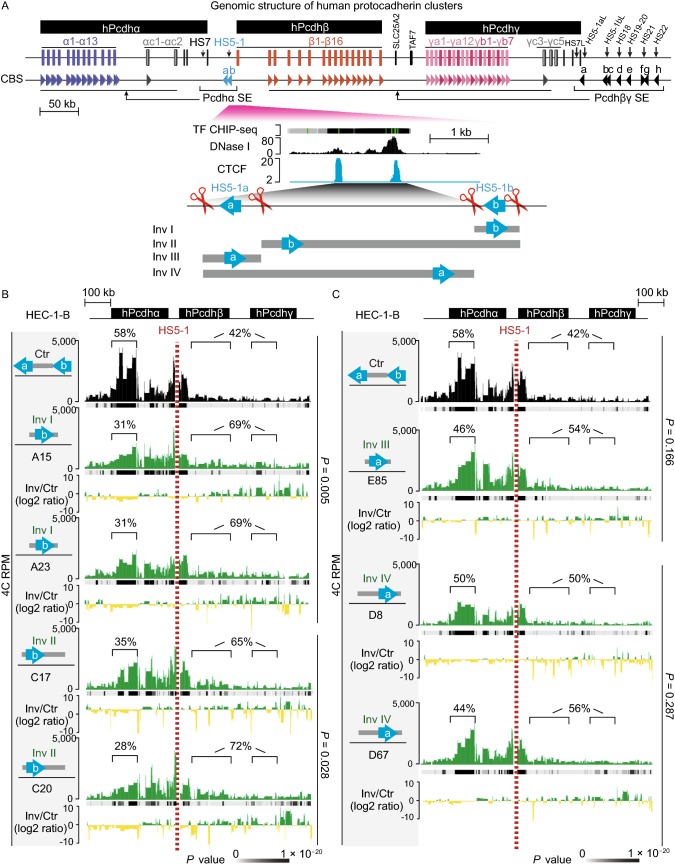

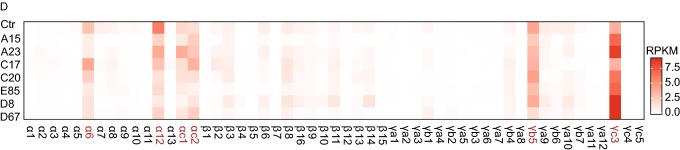
**Genetic dissection of the*Pcdhα HS5-1*enhancer architecture by CRISPR DNA-fragment editing.** (A) Schematics of the three human *Pcdh* gene clusters (hPcdh *α*, *β*, and *γ*) and the inversion experiments mediated by CRISPR DNA-fragment editing. The orientation of CBS arrays is indicated by horizontal arrowheads. The DNase I hypersensitive sites within super-enhancers are indicated by vertical arrows. The *HS5-1* enhancer and its various CRISPR inversions are highlighted below. Two tandem CBSs in a reverse orientation (*HS5-1a* and *HS5-1b*) at the editing sites are also highlighted. SLC25A2 and TAF7 are two non-Pcdh genes. CBS, CTCF-binding site; HS, hypersensitive site; SE, super-enhancer; TF, transcription factor. (B) Shown are 4C chromatin-interaction profiles in the wild-type control (Ctr) and CRISPR cell lines with inversion of the CBS *HS5-1b* only (Inv I) or the *HS5-1b* combined with the middle region (Inv II) using the *HS5-1* enhancer as a viewpoint. The significance of interactions (*P* value) is shown under the read’s density. The log2 ratios between each inversion cell clone and wild-type cells are also indicated. (C) Shown are 4C chromatin-interaction profiles in wild-type control and CRISPR cell lines with inversion of the CBS *HS5-1a* only (Inv III) or the *HS5-1a* combined with the middle region (Inv IV) using the *HS5-1* enhancer as a viewpoint. (D) Heatmap shows the alteration of expression patterns of members of the three *Pcdh* gene clusters resulting from CRISPR inversion of different enhancer and insulator (CBS) elements by RNA-seq experiments

Initial bioinformatics analysis revealed that, upstream of each variable-exon coding region (except *αc2*, *β1*, *γc4*, and *γc5*) of the *Pcdh α*, *β*, and *γ* gene clusters, there is a “CGCT” box-containing conserved sequence element (*CSE*) (Fig. S1B–D) (Wu et al., 2001). *CSE* is a forward-oriented CBS (CTCF-binding site) whose methylation on the “CGCT” box precludes the binding of CTCF proteins (Guo et al., 2012; Monahan et al., 2012). In addition, there is an exonic CBS (*eCBS*) within each variable-exon coding region of each member (except *αc1* and *αc2*) of the *Pcdhα* gene cluster (Fig. S1E). The downstream regulatory regions of the *Pcdhα* cluster were initially identified as clusters of DNase I hypersensitive sites (*HS1-15*) and function as strong enhancers by a luciferase reporter in transgenic mice and by targeted deletion in ES cells (Ribich et al., 2006), thus hereafter referred as *Pcdhα* super-enhancer. The five distal clustered hypersensitive sites (*HS5-1*) are flanked by two reverse-oriented CBSs (*HS5-1a* and *HS5-1b*) (Figs. S1F, S2A, and S2B) (Guo et al., 2012; Mountoufaris et al., 2018). The one proximal hypersensitive site (*HS7*) is not associated with any CBS (Fig. S2A and S2B). *In situ* CRISPR inversion of the *HS5-1* enhancer demonstrated that its activity is not orientation-independent in the native chromosomal context *in vivo* (Guo et al., 2015), despite overwhelming evidence that enhancers function in an orientation-independent manner in reporter assays *in vitro.*

To further investigate the role of enhancers and insulators as well as their locations and relative orientations in 3D genome architecture and gene regulation, we designed a series of dual sgRNAs to dissect the *HS5-1* enhancer architecture (Fig. 1A) (Shou et al., 2018). We made use of human HEC-1-B cells and screened single-cell CRISPR clones for inversion of the single CBS *HS5-1a* or *HS5-1b* site as well as its combination with the middle enhancer region of the *HS5-1* super-enhancer (Fig. S3). Remarkably, inversion of the CBS *HS5-1b* only or with the middle enhancer region results in a significant decrease of long-distance DNA-looping interactions between the *HS5-1* enhancer and *Pcdhα* promoters, and a corresponding increase of long-distance DNA-looping interactions with the *Pcdhβγ* promoters (Fig. 1B). However, inversion of the CBS *HS5-1a* only or combined with the middle enhancer region results in no significant alteration of long-distance DNA-looping interactions with either the *Pcdhα* or *Pcdhβγ* promoters (Fig. 1C). Thus, the relative orientations of the single CBS *HS5-1b* at the *Pcdhα* chromatin domain CCD or subTAD boundary determine the chromatin-looping directions between the distal enhancer and its target promoters.

To assess functional consequences of the altered chromatin looping, we performed RNA-seq experiments and found that each CRISPR inversion cell line with disrupted architecture of the *HS5-1* enhancer displays altered patterns of *Pcdh* gene expression (Fig. 1D). Finally, a series of CRISPR inversions of progressive numbers of CBSs in the *β-globin* locus demonstrate that only those inversions covering the CBS at a TAD boundary (CBS15) switch chromatin-looping directions (Figs. S4 and S5). We conclude that single CBSs at domain boundaries determine chromatin looping orientation and function as insulators.

The super-enhancer of the *Pcdh βγ* clusters, which is located downstream of *Pcdhγ* cluster, contains tandem reverse CBSs and one forward CBS (Figs. 1A, S1A and S1F) (Yokota et al., 2011; Guo et al., 2015). We analyzed the published transcription-factor binding landscape in the *Pcdhβγ* regulatory region of SK-N-SH cells and found that it is enriched with high levels of active marks of P300, Pol2, and H3K4me3, as well as CTCF and its associated cohesin complex (Fig. S2C) (Guo et al., 2015). Similar to the SK-N-SH cells, this region is enriched with high levels of P300, Pol2, H3K4me1, H3K27ac, and CTCF, as well as Med1, Oct4, Sox2, and Nanog in mouse ES cells (Fig. S2D). Moreover, this region is also marked with H3K27ac and H3K4me1 in mouse brain tissues, suggesting that it is an active super-enhancer *in vivo* (Guo et al., 2015). Finally, systematic characterization of the region revealed specific enrichments of CTCF proteins. Together, this suggests that the CTCF-enriched regulatory region is a super-enhancer regulating the 3D chromatin architecture of the *Pcdhβγ* clusters.

Next, we made use of the murine neuroblastoma cell line Neuro2A to investigate the chromatin architecture of the super-enhancer and its regulation of the *Pcdhβγ* promoters. We performed RNA-seq and circularized chromosome conformation capture (4C) experiments and found that *HS7L*, *HS5-1bL*, and *HS18-20* form specific long-distance chromatin interactions with the promoter regions of the expressed *Pcdhβγ* genes in Neuro2A cells (Fig. 2A and 2B). These results indicated that the active *Pcdhβγ* promoters, for example *Pcdh β11*/*12*, *β17*, *β22*, *γb1*, *γb8*, and *γc3*, are in spatial contacts with the super-enhancer (Fig. 2A and 2B).

**Figure 2:**
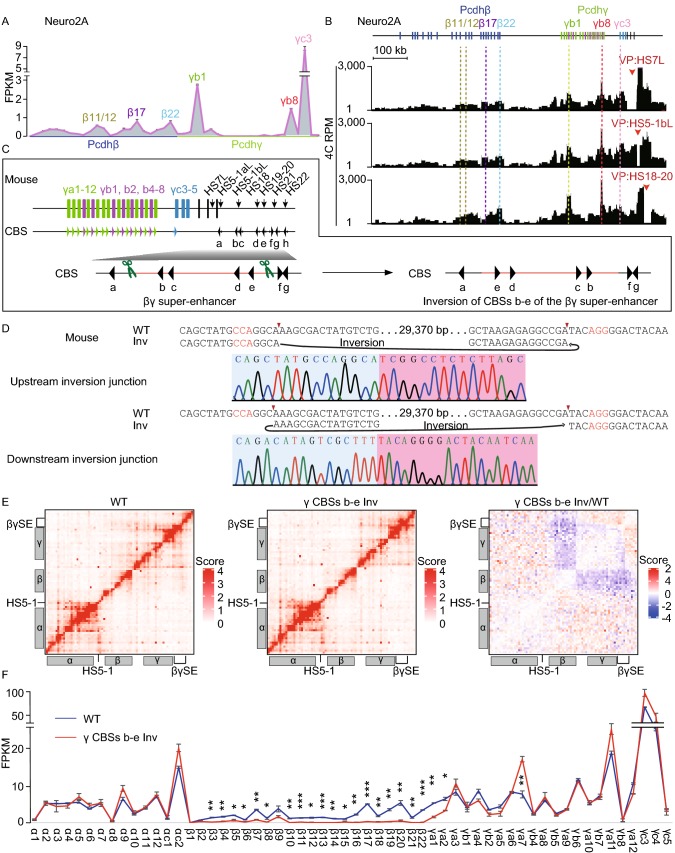

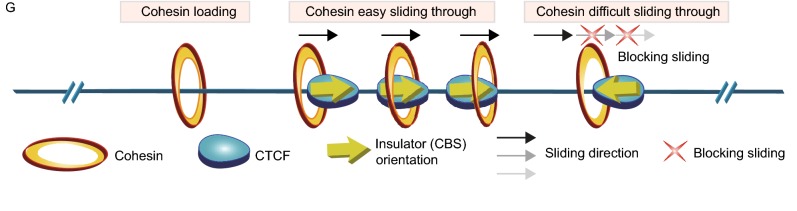
**Genetic dissection of the super-enhancer of the*Pcdhβγ*clusters by CRISPR DNA-fragment editing.** (A) RNA-seq results of the expression of distinct isoforms of the *Pcdhβγ* clusters in mouse Neuro2A cells. (B) The 4C interaction profiles of distinct isoforms of the *Pcdhβγ* clusters with the *HS7L*, *HS5-1bL*, or *HS18-20* as a viewpoint. (C) Schematic showing the dual-sgRNAs-mediated DNA-fragment inversion of the super-enhancer CBSs *b-e* in mice *in vivo*. Note the upstream staggered cleavage at − 4 positon on the noncomplementary strand by Cas9. (D) Confirmation of upstream and downstream junctions of the CBSs *b-e* inversion mice by Sanger sequencing. (E) 5C heatmap of the entire *Pcdh* locus in P0 mouse brain tissues. (F) RNA-seq showing a significant decrease of expression levels of members of the *Pcdhβ* cluster. Data are means ± SEM (*n*  = 2). *P  < 0.05, **P  < 0.01, ***P  < 0.001. (G) An asymmetric blocking model of cohesin sliding by directional CTCF binding to oriented insulators as suggested by genetics data

To study how the super-enhancer downstream of the *Pcdhγ* cluster regulates the higher-order chromatin architecture of the *Pcdhβγ* clusters *in vivo*, we generated the super-enhancer CBSs *b-e* inversion CRISPR mice by DNA-fragment editing (Fig. 2C and 2D). We then performed 5C (chromosome conformation capture carbon copy) and RNA-seq experiments using the CBSs *b-e* inverted mouse brain tissues and found that there is a significant decrease of long-distance chromatin interactions between the super-enhancer and members of the *Pcdhβ* cluster (Fig. 2E). Consistent with the 5C experiments, RNA-seq experiments revealed that there is a significant decrease of expression levels of the *Pcdhβ* genes (Fig. 2F). Intriguingly, the expression levels of *Pcdhγa7* are significantly increased (Fig. 2F).

The variable regions of the three *Pcdh* clusters contain forward-oriented tandem arrays of CBSs (Fig. S1A–E). To investigate the roles of these variable-region CBSs in the regulation of higher-order chromatin structure of the clustered *Pcdh* genes, we generated large deletions of forward-oriented tandem-arrayed CBSs by Cre/*LoxP*-mediated site-specific *trans*-allelic recombination in mice *in vivo* (Fig. S6A). Specifically, we bred mice to generate *delα* and *delαβ* mouse lines with the deletion of 24 or 45 forward-oriented tandem CBSs, respectively, but with the *Pcdhα1 CSE* intact (Fig. S6B and S6C). Conformation capture 4C experiments with *HS5-1*, *γa3*, or *HS5-1bL* as a viewpoint (VP) revealed that there is a significant increase of long-distance chromatin interactions between the viewpoint and *Pcdhα1 CSE* (Fig. S6D–F), suggesting that these large tandem-arrayed promoter CBSs function as insulators for the *Pcdhα1* promoter. By contrast, there appears no significant alteration of long-distance chromatin interactions between the viewpoint and CBSs located downstream of the large deletions (Fig. S6E and S6F). These data demonstrated that the tandem-arrayed promoter CBSs function as insulators and that these insulators alter long-distance chromatin interactions of upstream but not downstream CBSs, thus functioning in an asymmetric manner *in vivo*.

To investigate whether the insulation activity of CBSs is orientation-dependent *in vitro*, we generated a series of luciferase reporter constructs (Fig. S6G). Interestingly, the pair of tandem CBSs in the forward-reverse orientation flanking the luciferase reporter has the strongest enhancer-blocking activity (Fig. S6H). This demonstrates that CBSs function as enhancer-blocking insulators in an orientation-dependent manner in the luciferase reporter assay *in vitro*, similar to functioning in the none-orientation-independent manner *in vivo*.

Recent studies revealed that the locations and relative orientations of CBSs genome-wide determine the 3D chromosomal architecture (Rowley and Corces, 2018). In particular, the orientation and relative locations of a repertoire of *Pcdh* and *β-globin* CBSs determine the directional looping between the distal enhancer and its target promoters (Guo et al., 2015). The *HS5-1* enhancer is flanked by two reverse CBSs (Fig. 1A) and there is no evidence to date that single CBS sites can determine chromatin-looping directions between enhancers and promoters. To this end, we inverted reverse-oriented single CBSs at chromatin domain boundaries and found that single CBSs determine the direction of chromatin looping in the *Pcdh* and *β-globin* gene clusters (Figs. 1 and S4). In addition, deletion of large regions containing forward-oriented tandem-CBS arrays in mice *in vivo* demonstrated asymmetric influences of chromatin looping between super-enhancers and target promoters (Fig. S6). Finally, our CBSs *b-e* inversion in mice *in vivo* is consistent with previous deletion experiments that this region mainly enhances promoter activities of members of the *Pcdhβ* cluster (Yokota et al., 2011). Thus, the super-enhancer is pivotal to *Pcdhβ* expression and both deletion and inversion of its CBSs abolish long-distance chromatin looping to the distal *Pcdhβ* cluster beyond the proximal *Pcdhγ* cluster. In addition, the super-enhancer also regulates members of the *Pcdhγ* cluster by forming asymmetric chromatin looping interactions (Figs. 2B, 2E, S6E, and S6F). We proposed an asymmetric blocking model of cohesin sliding by insulator-bound CTCF proteins (Fig. 2G). Further experiments are needed to systematically dissect regulatory mechanisms and 3D chromatin architectures of the *Pcdhβγ* clusters.

## Electronic supplementary material

Below is the link to the electronic supplementary material.

Supplementary material 1 (PDF 3375 kb)
