# Supplementary material for: Genetic evidence for asymmetric blocking of higher-order chromatin structure by CTCF/cohesin
Source: Protein Cell. 2019 Sep 26;10(12):914–20. doi: 10.1007/s13238-019-00656-y (PMC6881428; doi:10.1007/s13238-019-00656-y)
Supplement: Supplementary file 1 — Supplementary material 1 (PDF 3375 kb) [file 13238_2019_656_MOESM1_ESM.pdf]

Supplementary Materials, including 6 Figures and one Table as well as Materials and methods

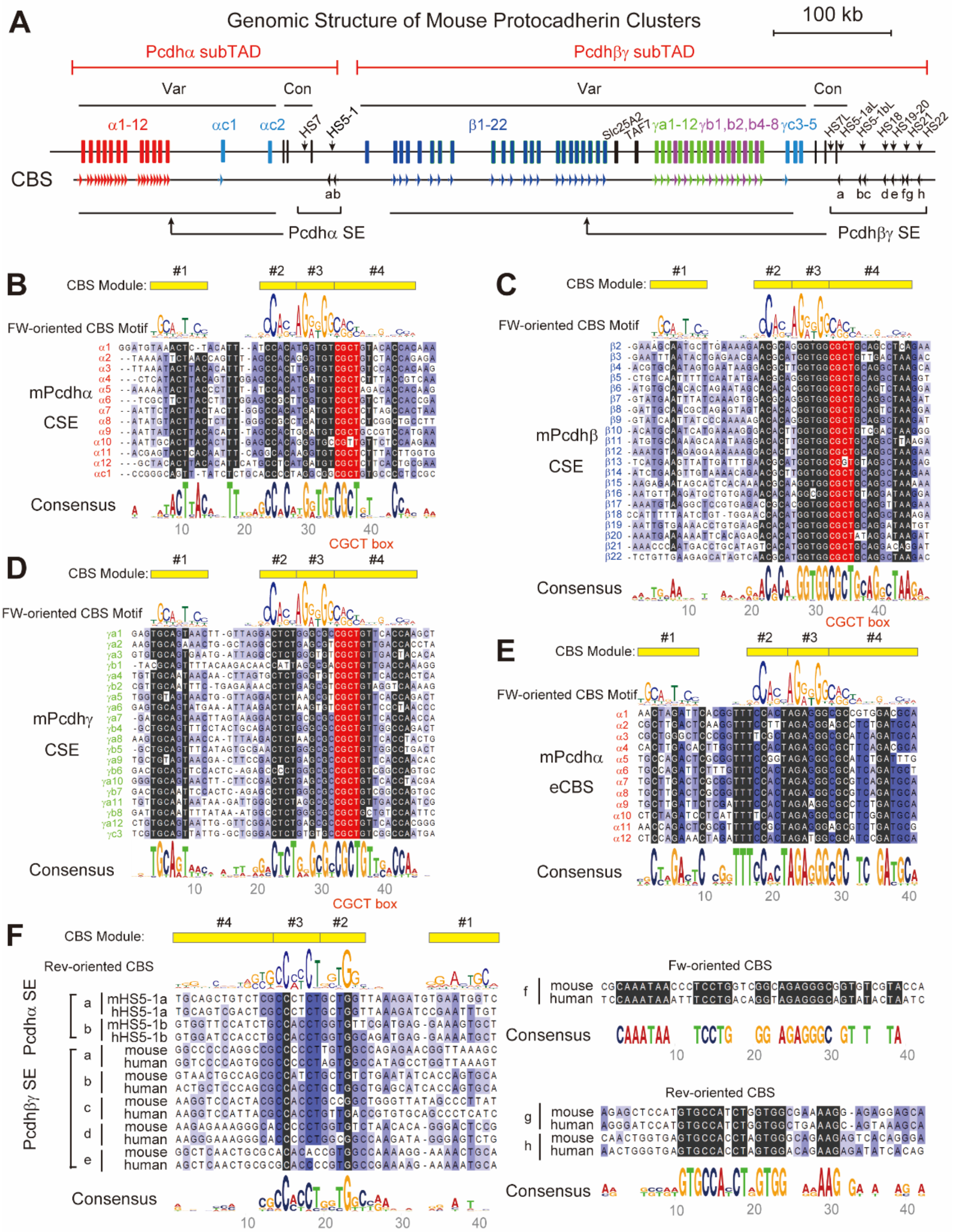

Fig. S1. Genomic structure and distribution of CTCF sites within the mouse *Pcdh* α, β, and γ gene clusters. (**A**) Genomic structure of the mouse *Pcdh* α, β, and γ gene clusters. The CTCF binding sites (CBSs) and the DNase I hypersensitive sites (HS) are indicated as horizontal arrowheads below and vertical arrows above, respectively. SE: super-enhancer. (**B-D**) The conserved

sequence element (CSE) upstream of each variable-exon-coding region of members of the mouse *Pcdh* α (**B**), β (**C**), and γ (**D**) cluster (except α*c2*, β*1*, γ*c4*, and γ*c5*) contains a "CGCT" box. These sequence elements are CBSs in the forward (FW) orientation corresponding to the direction of the CBS modules 1-4. The CGCT box is highlighted on a red background. mPcdh: mouse protocadherin. (**E**) An exonic CTCF binding site (eCBS) within the variable-exon coding region of each *Pcdh*α gene is located at about 1 kb downstream of the respective CSE. (**F**) The sequences of CBS modules 4-1 (reverse orientation) within the DNase I hypersensitive sites in the *Pcdh* α and $\beta\gamma$ super-enhancers.

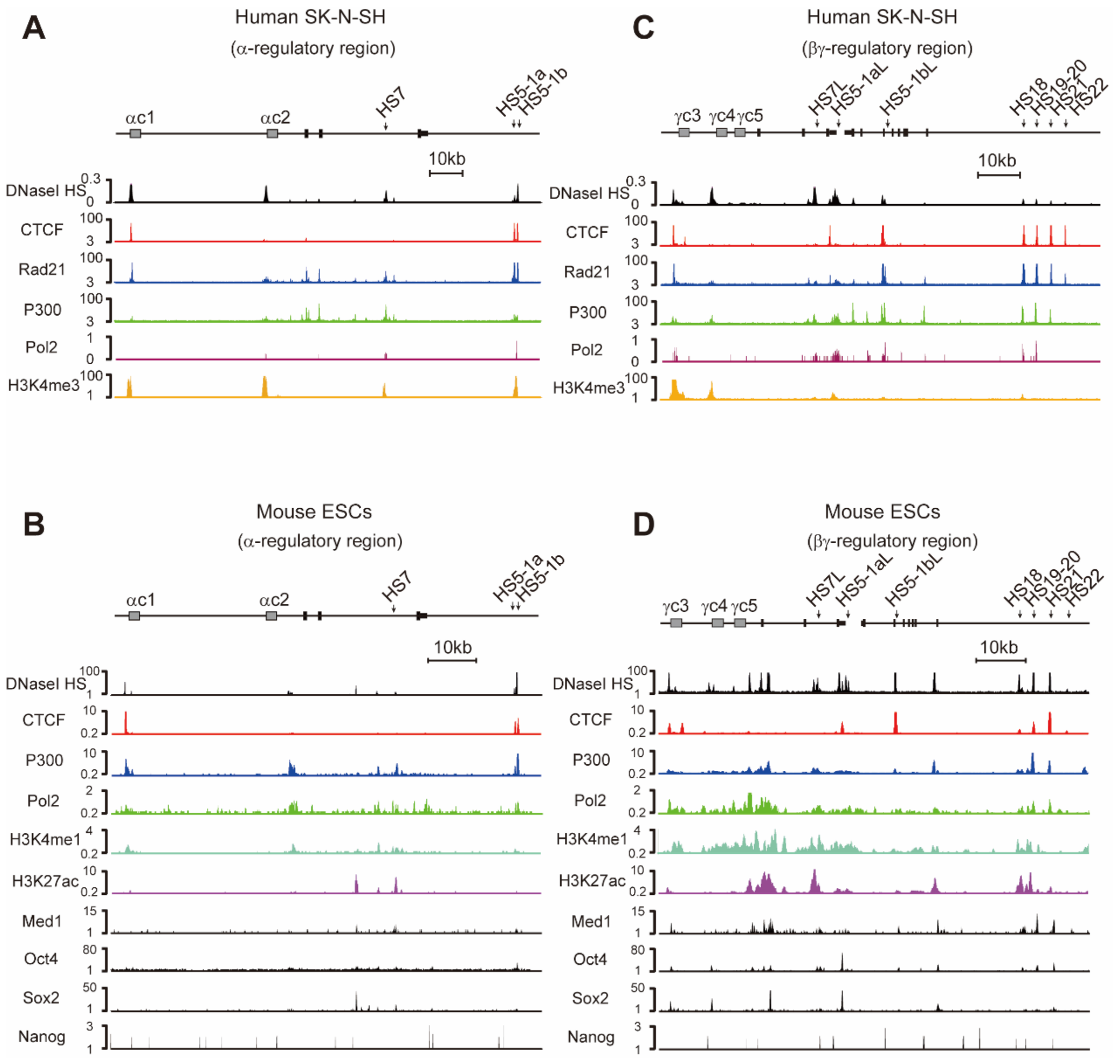

Fig. S2. Molecular marks in the *Pcdh* α and $\beta\gamma$ super-enhancers. (**A,B**) The signal profiles of ChIP-seq in the regulatory region downstream of the *Pcdh*α in the human SK-N-SH (**A**) and mouse ES cells (**B**) (ENCODE Project Consortium, 2012; Guo et al., 2012). (**C,D**) The signal profiles of ChIP-seq in the regulatory region downstream of the *Pcdh*$\beta\gamma$ clusters in the SK-N-SH (**C**) and mouse ES cells (**D**) (ENCODE Project Consortium, 2012; Shen et al., 2012). The locations of DNase I hypersensitive sites are indicated by vertical arrows.

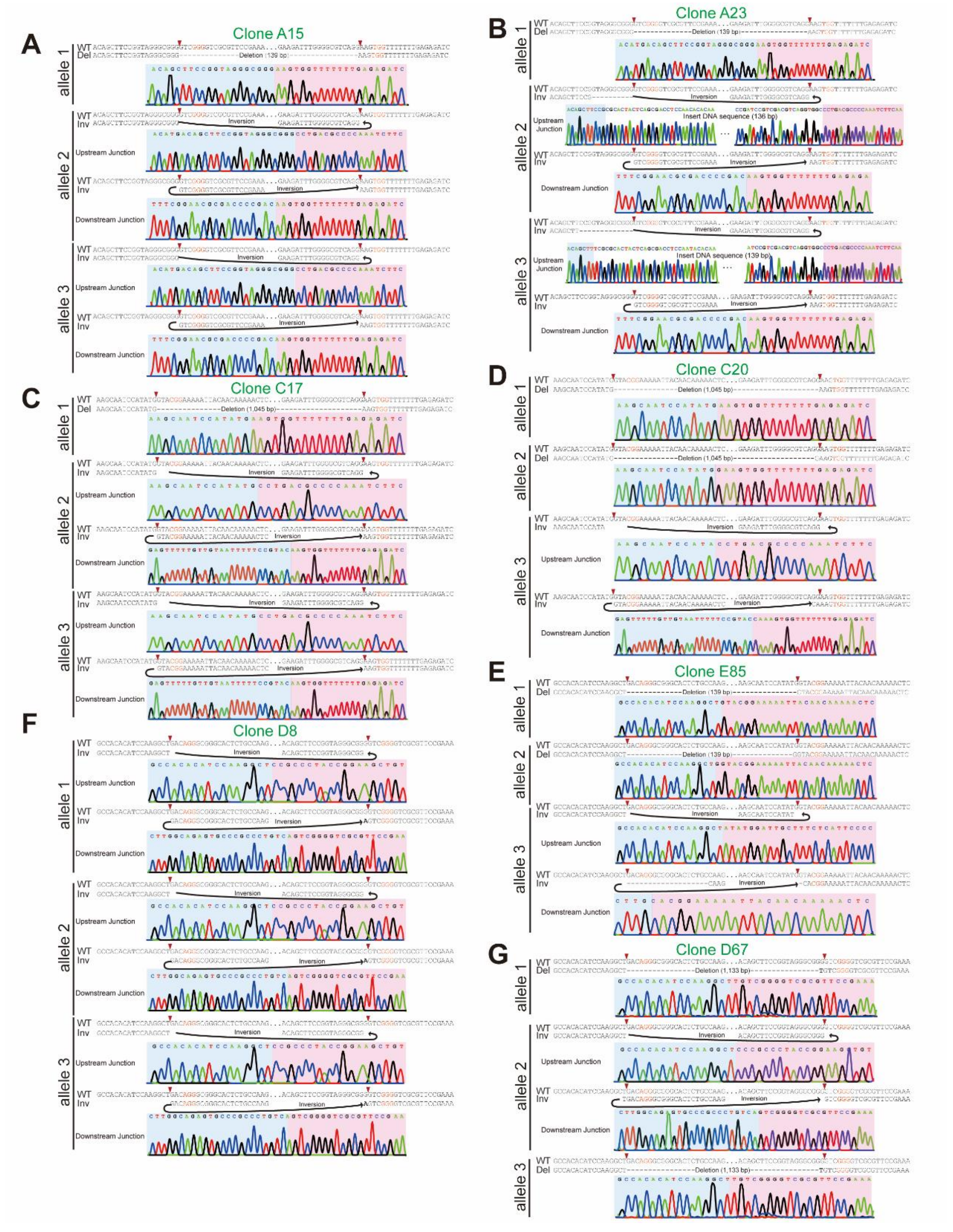

Fig. S3. Genotyping of CRISPR inversion cell clones of the *Pcdh* gene clusters. Sanger sequencing of the three alleles of the junctions of inversion or deletion. Clone A15 (**A**) and Clone A23 (**B**) for Inv I. Clone C17 (**C**) and Clone C20 (**D**) for Inv II. Clone E85 (**E**) for Inv III. Clone D8 (**F**) and Clone D67 (**G**) for Inv IV. Inv, inversion; Del, deletion.

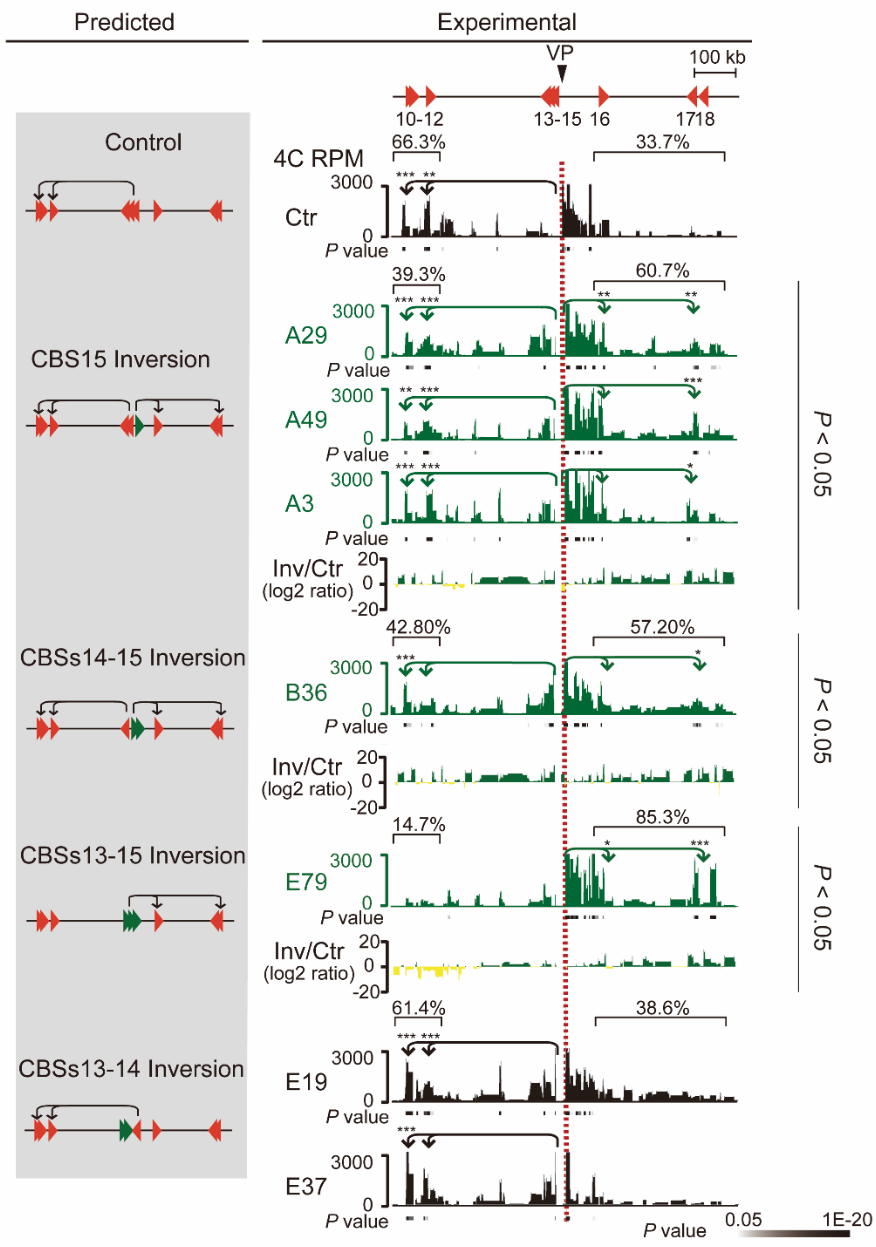

Fig. S4. DNA-fragment inversion of progressive numbers of tandem CBS sites at the boundary of the *β-globin* chromatin domain. Shown are the schematics of predicted long-distance chromatin interactions by inverting the relative orientation of CBSs in the *β-globin* locus. The chromatin-interaction profiles in control and various CBSs inversion clones using CBSs13-15 as a viewpoint (VP) are shown in the right panel. The CBSs13-15 cell clone (E79) is a positive control. Note that the inversion of the two internal CTCF sites of CBSs13-14 does not switch the chromatin-looping direction. Only inversions covering the boundary CBS15 switch the chromatin-looping direction. The significance of interactions (*P* value) is shown under the read's density. The log2 ratios between inversion and control clones are also indicated. **P* <0.05, ***P* < 0.01, ****P* < 0.001.

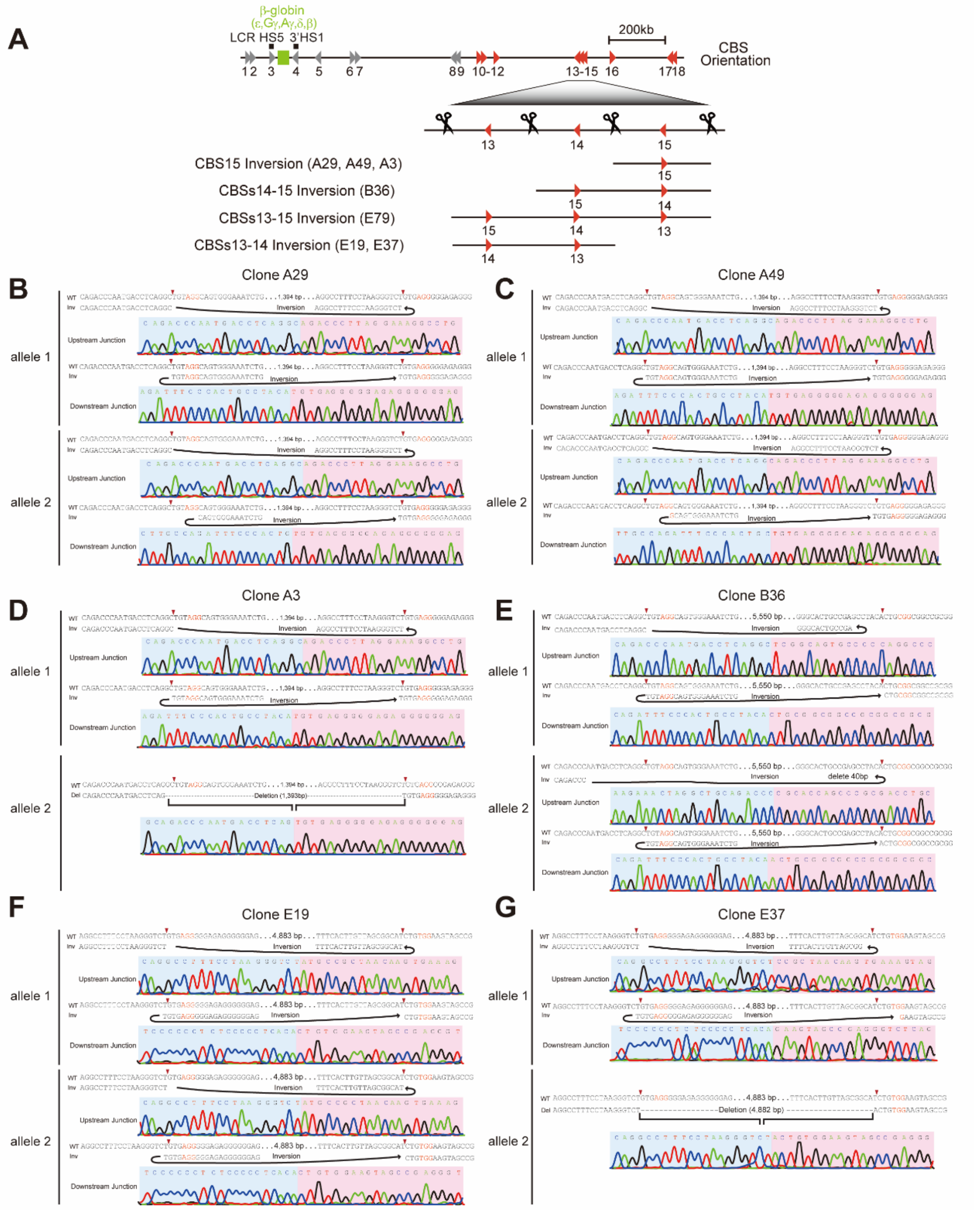

Fig. S5. Genotyping of CRISPR inversion cell clones of the *β-globin* locus. (**A**) Schematic of the oriented CBS clusters and the various DNA-fragment inversions of CBS15, CBSs14-15, or CBSs13-14 induced by Cas9 with dual sgRNAs in the *β-globin* locus. (**B-G**) DNA sequencing results of the two alleles show the junctions of inversion or deletion. Clone A29 (**B**), Clone A49 (**C**), and clone A3 (**D**) for CBS15 Inversion. Clone B36 (**E**) for CBSs14-15 Inversion. Clone E19 (**F**) and Clone E37 (**G**) for CBSs13-14 Inversion.

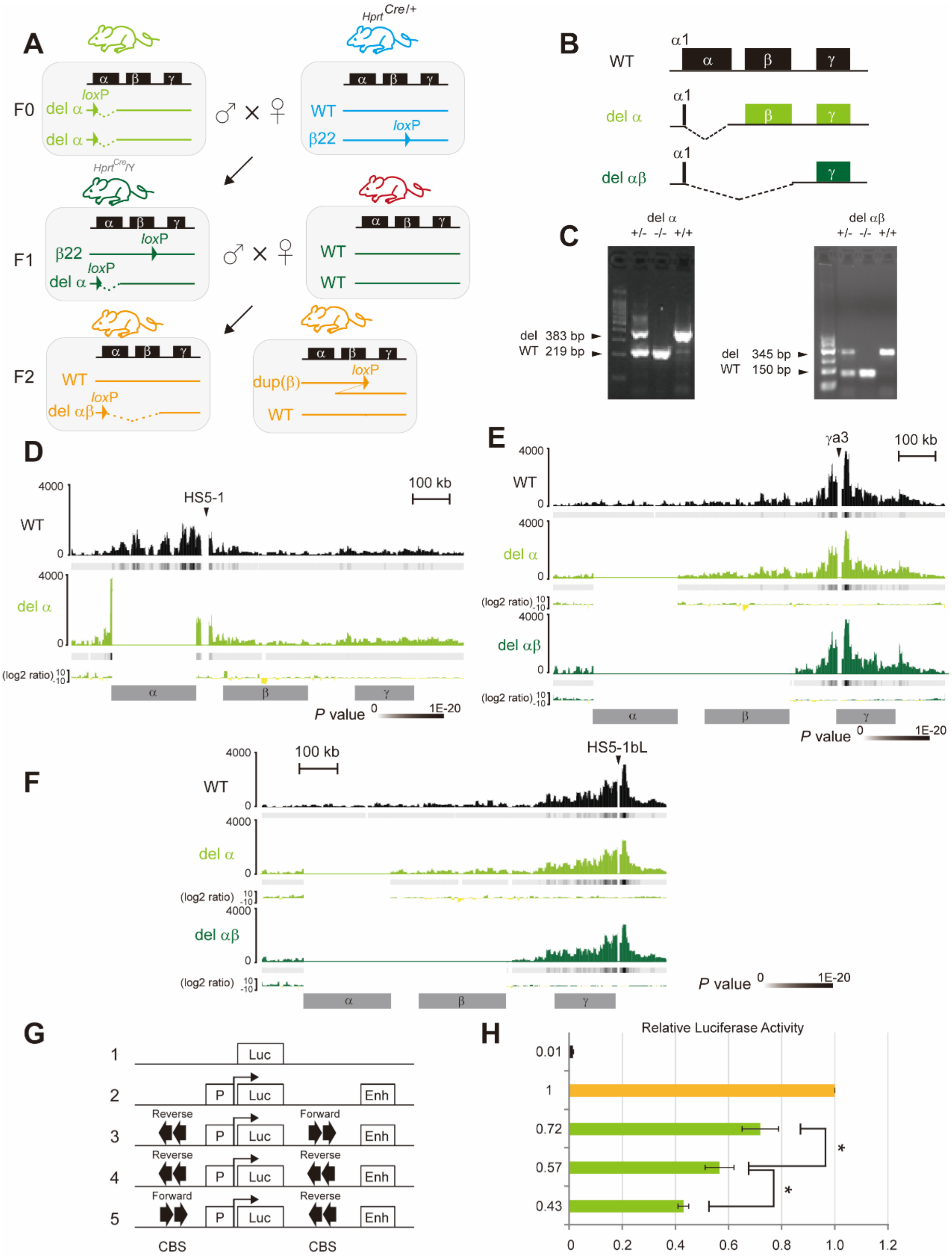

Fig. S6. Genetic dissection of the *Pcdh* promoter regions. (A) Diagram showing the strategy for generating *Pcdhαβ* double knockout mice by *trans*-allelic recombination in compound heterozygous mice obtained by crossing the *α* cluster-deletion of 24 CBSs with the *β22*-deletion of 21 CBSs as well as *Hprt-Cre* mice. (**B, C**) Diagram and genotyping of the 24-CBSs-*Pcdhα* or 45-CBSs-*Pcdhαβ* deletion mice. (**D-F**) 4C interaction profiles with *HS5-1* (**D**), *γa3* (**E**), *HS5-1bL* (**F**) as a viewpoint of mouse brain tissues with targeted deletion of the *Pcdhα* or *Pcdhαβ* clusters of 24 or 45 forward-oriented CBSs, respectively. (**G,**

H) Enhancer-blocking activity measured by luciferase (Luc) reporter assays. Pairs of tandem CBSs located between enhancer (Enh) and promoter (P) as well as upstream of promoter in three different configurations of CBS orientations. The basic vectors were used as controls. Data are means ± SEM (n=4). **P* <0.05.

Materials and methods

Animals. The generation of the *Pcdh*α cluster deletion mice was previously described (Lu et al., 2018; Suo et al., 2012; Wu et al., 2007, 2008). The *Pcdh*$\alpha\beta$ double knockout mice were generated from Cre/*loxP*-mediated *trans*-allelic recombination in compound heterozygous mice obtained by crossing the α-cluster-deletion with β*22*-deletion as well as *Hprt*-*Cre* mice (Wu et al., 2007, 2008) (Fig. S6A). The CBSs *b*-*e* inversion mice were generated by microinjection of zygotes with Cas9 mRNA and a pair of sgRNAs. Primers for genotyping are listed in Supplementary Table S1. Animal experiments were approved by the Institutional Animal Care and Use Committee (IACUC) of Shanghai Jiao Tong University.

Cell culture. Human HEK293T cells were cultured in DMEM (HyClone) supplemented with 10% FBS (Gibco) and 1% penicillin–streptomycin (Gibco). Human HEC-1-B cells and mouse Neuro2A cells were cultured in MEM (Gibco) supplemented with 10% FBS, 1% penicillin–streptomycin, 2 mM glutamine (Gibco), and 1 mM sodium pyruvate (Sigma). Cells were cultured at 37°C in a humidified incubator containing 5% CO_2 and passaged every two or three days. Cells were plated at a density of approximately 4×10^5 cells in each well of 12-well plates and transfected 24 hours later.

Single-cell screening of DNA-fragment inversions by CRISPR. CRISPR single-cell inversion clones were screened as previously described (Guo et al., 2015; Li et al., 2015). For the *Pcdh* locus, we obtained 2 (A15 and A23), 1 (E85), 2 (C17 and C20), and 2 (D8 and D67) inversion HEC-1-B clones of CBS *HS5-1b* and *HS5-1a* as well as their combination with the middle enhancer regions from 44, 85, 67, and 77 single-cell clones, respectively. For the *β-globin* locus, we obtained 3 (A3, A29, and A49), 1 (B36), 2 (E19 and E37) inversion HEK293T clones of CBS15, CBSs14-15, and CBSs13-14 from 49, 40, and 40 single-cell clones, respectively.

Comparative Sequence Analysis. DNA sequences were aligned with ClustalW (Larkin et al., 2007) by default parameters and were visualized by JalView (Waterhouse et al., 2009). WebLogo was used to make sequence motifs (Crooks et al., 2004).

Circularized chromosome conformation capture (4C). 4C experiments were performed as previously described (Guo et al., 2015; Jia et al., 2014; Simonis et al., 2006; Zhao et al., 2006) with some modifications. Briefly, P0 mouse brain tissue was digested with 0.625 μg/ml collagenase in the DMEM supplemented with 10% FBS for 45 min at 37°C. After gently pipetting, dispersed cells were filtered through a 40 μm cell strainer (BD Biosciences) to make single-cell

suspension. For human cell lines, cells were digested with trypsin to make single-cell suspension. A total of 10^7 cells were used for each experiment. After crosslinking, cells were permeabilized and digested with HindIII overnight at 37°C. The digested nuclei were then ligated with T4 DNA ligase. Ligated DNA was then extracted and digested with a second enzyme (DpnII or NlaIII) and ligated again. Finally, 4C-seq library was generated by PCR (Primers are listed in Supplementary Table S1). High-throughput sequencing was performed on Illumina HiSeq X Ten platform. Reads were mapped to human GRCh37/hg19 or mouse mm9 reference genomes using the Bowtie program (version 1.0.0) (Langmead and Salzberg, 2012).The r3Cseq program in the R/Bioconductor package (Thongjuea et al., 2013) was used to detect statistically significant long-range chromatin-looping interactions.

CRISPR/Cas9 system. The templates for producing targeting sgRNAs were constructed by PCR using the pGL3-U6-sgRNA-PGK-Puro plasmid (Li et al., 2015; Shou et al., 2018) with appropriate primers (Supplementary Table S1). All plasmids were confirmed by sequencing.

Luciferase reporter assay. Luciferase reporter constructs were modified from the pGL3 vector from Promega. A multiple cloning site containing EcoRI and SacI sites was inserted downstream of the firefly luciferase reporter gene using

PCR (Primers are listed in Supplementary Table S1). The SV40 enhancer was cloned downstream of the two restriction enzyme sites. CTCF-binding sequences were inserted upstream of the SV40 promoter, and between the firefly luciferase reporter gene and the SV40 enhancer to generate a series of luciferase reporter constructs. All of the constructed plasmids were confirmed by sequencing. The constructs were linearized by NotI digestion before transfection. HEK293T cells at about 40% confluence were transfected with 200 ng of plasmid DNA using the Lipofectamine 2000 reagents (Invitrogen) in a 96-well plate. Firefly and Renilla luciferase activities were assayed 48 hours after transfection using the Dual Glo reagent (Promega) and a Synergy 2 Microplate Reader (BioTek). The experiments were performed four times using independent DNA preparations.

***In-vitro* transcription of Cas9 mRNA and sgRNA.** To obtained Cas9 mRNA, Cas9 vector (Chang et al., 2013) was first linearized with XbaI and then transcribed with T7 polymerase by an mRNA *in-vitro* transcription Kit according to the manufacturer's guide (Life Technologies). SgRNAs were obtained by PCR amplifications (Primers are listed in Supplementary Table S1) and then *in-vitro* transcribed with MEGAshortscript Kit (Life technologies). Cas9 mRNA and sgRNAs were purified with MEGAclear Kit (Life technologies) and dissolved in TE buffer for microinjections.

One-cell embryo injection. All animal procedures were performed according to Institutional Animal Care and Use Committee of Shanghai Jiao Tong University. C57BL/6 and ICR female mice were used as embryo donors and foster mothers, respectively. Super ovulated female C57BL/6 mice (about 7 weeks old) were mated to stud males, and fertilized embryos were collected from oviducts 20 hours later. Cas9 mRNA (100 ng/μl) and sgRNA (50 ng/μl) were injected into the cytoplasm of fertilized eggs with well recognized pronuclei in M2 medium (Sigma). The injected embryos were cultured in KSOM medium (Millipore) for 4 days at 37°C in a 5% CO_2 incubator. The survivors of the injected embryos were implanted into the oviducts of pseudo-pregnant ICR females.

Chromosome conformation capture-carbon copy (5C) primer design. 80 forward and 80 reverse primers covering the *Pcdh gene* clusters were designed by My5C tools (http://my5c.umassmed.edu). Primers were designed to recognize 3'-end of HindIII restriction fragments of either the Watson or Crick strand. All forward primers contain CGG at 5'-end and a modified T7 universal primer sequence (TAATA CGACT CACTA TAGCC) followed by a unique sequence and a half of the HindIII restriction site (AAG). All reverse primers contain a half of the HindIII restriction site (CTT) at 5'-end followed by a unique

sequence and a modified complementary T3 universal sequence (TCCCT TTAGT GAGGG TTAAT A) and 5'-3' TGC. All 5C primers are shown in Supplementary Table S1.

5C library preparation. Six bacterial artificial chromosomes (BACs) covering the *Pcdh* gene clusters were mixed, digested with HindIII and randomly ligated as the 5C control library for normalization. Chromosome conformation captured (3C) library was generated as described before (Guo et al., 2012). The concentration of the prepared 3C library was determined using the Picogreen dsDNA quantitation assay. The prepared 3C library (about 500 ng) and control BAC library (5 ng) was mixed with 1 μg and 1.5 μg Salmon Testis DNA (Sigma, cat no. D7656-1ML), respectively. Each sample was then mixed with 1.7 fmol of each 5C primer and 1 μl 10 x 5C annealing buffer (20 mM Tris-acetate pH7.9, 50 mM potassium acetate, 10 mM magnesium acetate, 1 mM DTT) and water to a total volume of 10 μl. The samples were denatured for 5 min at 95°C followed by an annealing process of 16 h at 48°C. Then, each sample was mixed with 3 μl 10 x 5C ligation buffer (25 mM Tris-HCL pH7.6, 31.25 mM potassium acetate, 12.5 mM magnesium acetate, 1.25 mM NAD, 12.5 mM DTT and 0.125% (vol/vol) Triton X-100) and 10U Taq DNA ligase (NEB) and water to a total volume of 30μl. The ligation experiment was performed for 1h at 48°C and terminated by incubation for 10 min at 65°C. The ligated products were amplified by PCR to generate 5C libraries. The generated 5C libraries were

purified with MiniElute PCR Purification Kit (QIAGEN). The 5C PCR primers are listed in Supplementary Table S1.

5C sequencing data analysis. The 5C libraries were sequenced at the 90 bp pair-end mode with a HiSeq 2500 platform. The total numbers of reads which matches each forward and reverse primer pair (80 x 80) were counted. Different samples were normalized with the BAC control. The final heatmap was generated by two biological replicates.

RNA-seq. RNA-seq experiments were performed as previously described (Guo et al., 2015). Briefly, total RNA was extracted by Trizol reagents (Life Technologies) following the manufacturer's instructions. Library construction was started from 1 μg total RNA then selected by the oligo dT beads (NEB). RNA-seq libraries were generated using NEB Next Ultra™ RNA Library Prep Kit for Illumina (NEB #7530) following manufacturer's instructions. All RNA-seq experiments were performed with at least two biological replicates. RNA-seq libraries were sequenced on a HiSeq X Ten Platform. Sequenced reads were aligned to the reference genome using the TopHat software (v2.0.14) with default parameters. The expression levels of genes were measured using the Cufflinks software (v2.2.1) with default parameters.

References

Chang, N., Sun, C., Gao, L., Zhu, D., Xu, X., Zhu, X., Xiong, J.W., and Xi, J.J. (2013). Genome editing with RNA-guided Cas9 nuclease in zebrafish embryos. Cell Res. 23, 465-472.

Crooks, G.E., Hon, G., Chandonia, J.M., and Brenner, S.E. (2004). WebLogo: a sequence logo generator. Genome Res. 14, 1188-1190.

ENCODE Project Consortium (2012). An integrated encyclopedia of DNA elements in the human genome. Nature 489, 57-74.

Guo, Y., Monahan, K., Wu, H., Gertz, J., Varley, K.E., Li, W., Myers, R.M., Maniatis, T., and Wu, Q. (2012). CTCF/cohesin-mediated DNA looping is required for protocadherin alpha promoter choice. Proc. Natl. Acad. Sci. USA 109, 21081-21086.

Guo, Y., Xu, Q., Canzio, D., Shou, J., Li, J., Gorkin, D.U., Jung, I., Wu, H., Zhai, Y., Tang, Y., *et al.* (2015). CRISPR inversion of CTCF sites alters genome topology and enhancer/promoter function. Cell 162, 900-910.

Jia, Z., Guo, Y., Tang, Y., Xu, Q., Li, B., and Wu, Q. (2014). Regulation of the protocadherin *Celsr3* gene and its role in *globus pallidus* development and connectivity. Mol. Cell Biol. 34, 3895-3910.

Langmead, B., and Salzberg, S.L. (2012). Fast gapped-read alignment with Bowtie 2. Nat. Methods 9, 357-359.

Larkin, M.A., Blackshields, G., Brown, N.P., Chenna, R., McGettigan, P.A., McWilliam, H., Valentin, F., Wallace, I.M., Wilm, A., Lopez, R., *et al.* (2007). Clustal W and Clustal X version 2.0. Bioinformatics 23, 2947-2948.

Li, J., Shou, J., Guo, Y., Tang, Y., Wu, Y., Jia, Z., Zhai, Y., Chen, Z., Xu, Q., and Wu, Q. (2015). Efficient inversions and duplications of mammalian regulatory DNA elements and gene clusters by CRISPR/Cas9. J. Mol. Cell Biol. 7, 284-298.

Lu, W.C., Zhou, Y.X., Qiao, P., Zheng, J., Wu, Q., and Shen, Q. (2018). The protocadherin alpha cluster is required for axon extension and myelination in the developing central nervous system. Neural Regen. Res. 13, 427-433.

Shen, Y., Yue, F., McCleary, D.F., Ye, Z., Edsall, L., Kuan, S., Wagner, U., Dixon, J., Lee, L., Lobanenkov, V.V., *et al.* (2012). A map of the *cis*-regulatory sequences in the mouse genome. Nature 488, 116-120.

Shou, J., Li, J., Liu, Y., and Wu, Q. (2018). Precise and predictable CRISPR chromosomal rearrangements reveal principles of Cas9-mediated nucleotide insertion. Mol. Cell 71, 498-509.

Simonis, M., Klous, P., Splinter, E., Moshkin, Y., Willemsen, R., de Wit, E., van Steensel, B., and de Laat, W. (2006). Nuclear organization of active and inactive chromatin domains uncovered by chromosome conformation capture-on-chip (4C). Nat. Genet. 38, 1348-1354.

Suo, L., Lu, H., Ying, G., Capecchi, M.R., and Wu, Q. (2012). Protocadherin clusters and cell

adhesion kinase regulate dendrite complexity through Rho GTPase. J. Mol. Cell Biol. 4, 362-376.

Thongjuea, S., Stadhouders, R., Grosveld, F.G., Soler, E., and Lenhard, B. (2013). r3Cseq: an R/Bioconductor package for the discovery of long-range genomic interactions from chromosome conformation capture and next-generation sequencing data. Nucleic Acids Res. 41, e132.

Waterhouse, A.M., Procter, J.B., Martin, D.M., Clamp, M., and Barton, G.J. (2009). Jalview Version 2--a multiple sequence alignment editor and analysis workbench. Bioinformatics 25, 1189-1191.

Wu, S., Ying, G., Wu, Q., and Capecchi, M.R. (2007). Toward simpler and faster genome-wide mutagenesis in mice. Nat. Genet. 39, 922-930.

Wu, S., Ying, G., Wu, Q., and Capecchi, M.R. (2008). A protocol for constructing gene targeting vectors: generating knockout mice for the cadherin family and beyond. Nat. Protocol 3, 1056-1076.

Zhao, Z., Tavoosidana, G., Sjolinder, M., Gondor, A., Mariano, P., Wang, S., Kanduri, C., Lezcano, M., Sandhu, K.S., Singh, U.*, et al.* (2006). Circular chromosome conformation capture (4C) uncovers extensive networks of epigenetically regulated intra- and interchromosomal interactions. Nat. Genet. 38, 1341-1347.

Supplementary Table

Supplementary Table S1: Oligonucleotides Used.	
**Primer Name**	**5'-3' sequence**
**Primers used for genotyping (*Pcdh*α and *Pcdh*$\alpha\beta$ deletion mice)**	
ConF1	AGGCTGAATAACGTGCACAGCTAAG
ConR1	TGCAGATTGGTTCAATGGAGTCTTT
Beta22wtF	AGCTGAGCTACGAGTAGGAGACAT
GFPmutF	CCCCCTGAACCTGAAACATAAAATG
Beta22R	TGAACGCTGTTATTTCCCACATCC
**Primers used for genotyping (CRISPR *Pcdh*γ CBSs *b-e* inversion mice)**	
CTCF25-genotyping-F5	AGCCAGACCAGCATAGCAAAT
CTCF25-genotyping-F7	ACAGGATAATGGGTTCTGGAGC
CTCF25-genotyping-R3	TCCAGATTACGAGCTGAGCG
**Primers used for luciferase reporter assays**	
pGL3-re-R3 (MCS)	CGGGATCCGAGCTCGAATTCTTATCGATTTTACCACATTTGTAG
SV40enh F	CGGGATCCGAACGATGGAGCG
SV40enh R (SalI)	GTAGCCGTCGACGCTGTGGAATGTGTGTCAGTT
Forward-hPcdha8 F (KpnI)	GGGGTACCCGGAAGTAATTCATGTAATCATT
Forward-hPcdha8-HS5-1b R	CTATTGATAAAGTGTAAAGAACATTGACCAACCAGAAC
Forward-hPcdha8-HS5-1b F	GTTCTGGTTGGTCAATGTTCTTTACACTTTATCAATAG
Forward-HS5-1b R (XhoI)	GCCTCTCGAGGGTCGGGGTCGCGTTCC
Forward-hPcdha8 F (EcoRI)	GGAATTCCGGAAGTAATTCATGTAATC
Forward-HS5-1b R (SacI)	TGATCGAGCTCGGTCGGGGTCGCGTTCC

Reverse-hPcdha8 F (KpnI)	GGGGTACCAACATTGACCAACCAGAAC
Reverse-hPcdha8-HS5-1b R	GGAACGCGACCCCGACCCGGAAGTAATTCATGTAATC
Reverse-hPcdha8-HS5-1b F	GATTACATGAATTACTTCCGGGTCGGGGTCGCGTTCCGAAAAG
Reverse-HS5-1b R (XhoI)	GCCTCTCGAGCTTTACACTTTATCAATAGC
Reverse-hPcdha8 F (EcoRI)	GGAATTCAACATTGACCAACCAGAAC
Reverse-HS5-1b R (SacI)	TGATCGAGCTCCTTTACACTTTATCAATAGC
**Primers used for 5C-seq**	
5C_mPcdh_REV_1	CTTGCTGAACATGAGCTATCACCTGACTCTTCCCTTTAGTGAGGGTTAATATGC
5C_mPcdh_REV_2	CTTTTGGCCTTATGTCTAAGCATTTTCCACTCCCTTTAGTGAGGGTTAATATGC
5C_mPcdh_REV_3	CTTGAGAACCTCACTTCAGATCCCCAGTATTCCCTTTAGTGAGGGTTAATATGC
5C_mPcdh_REV_4	CTTCTTGCAGACTAGGCAAGCACTGTAGCTTCCCTTTAGTGAGGGTTAATATGC
5C_mPcdh_REV_5	CTTTGCCATTACTTCATTTTTAATGTCATCTCCCTTTAGTGAGGGTTAATATGC
5C_mPcdh_REV_6	CTTTTCTATTGCTAAAGTCTTCAACATTGATCCCTTTAGTGAGGGTTAATATGC
5C_mPcdh_REV_7	CTTCTGTACCAGTAAAATAAATAAATAAATTCCCTTTAGTGAGGGTTAATATGC
5C_mPcdh_REV_8	CTTTTGTTGAGTCGCTAAATATACTCAGGGTCCCTTTAGTGAGGGTTAATATGC
5C_mPcdh_REV_9	CTTGAAGGGGACATGAGGAGACAGGGACGTTCCCTTTAGTGAGGGTTAATATGC
5C_mPcdh_REV_10	CTTAAAGTTCTCACTAGTAAGCTGAATCATTCCCTTTAGTGAGGGTTAATATGC
5C_mPcdh_REV_11	CTTACTTCACAAAACTGGAAAATTTAAATGTCCCTTTAGTGAGGGTTAATATGC
5C_mPcdh_REV_12	CTTTGGCCGGGCACAACTGCTCCGCCAGGCTCCCTTTAGTGAGGGTTAATATGC

5C_mPcdh_REV_13	CTTTGTTTTACTTCCTTGATTTCTGTGTTCTCCCTTTAGTGAGGGTTAATATGC
5C_mPcdh_REV_14	CTTTCTAAGTGACAGCAATCTAAAACGTTTTCCCTTTAGTGAGGGTTAATATGC
5C_mPcdh_REV_15	CTTCGATGAAAATATAGAATACACCTATTTTCCCTTTAGTGAGGGTTAATATGC
5C_mPcdh_REV_16	CTTCCAAAAAACATTTTGACATCTTATGGTTCCCTTTAGTGAGGGTTAATATGC
5C_mPcdh_REV_17	CTTGGTGGATTGTCATTTATGTCAGAGATCTCCCTTTAGTGAGGGTTAATATGC
5C_mPcdh_REV_18	CTTAAATGTCTCTGCTGCTCCTTTATGACTTCCCTTTAGTGAGGGTTAATATGC
5C_mPcdh_REV_19	CTTCTCAATTTAAACAGCTGCTTCTGGGCTTCCCTTTAGTGAGGGTTAATATGC
5C_mPcdh_REV_20	CTTTGTGAACTTGGATTCAGAACTGAACGGTCCCTTTAGTGAGGGTTAATATGC
5C_mPcdh_REV_21	CTTACTCTGGGTCTGCAGCTCCAGTGGAACTCCCTTTAGTGAGGGTTAATATGC
5C_mPcdh_REV_22	CTTTAGCATGGTAATGTGGTGGGAACTGGTTCCCTTTAGTGAGGGTTAATATGC
5C_mPcdh_REV_23	CTTGTTAGCTGAGGCTAGTGCTCGCACTGGTCCCTTTAGTGAGGGTTAATATGC
5C_mPcdh_REV_24	CTTCACAGGCAGCCCGCACTCGTCCTCGATTCCCTTTAGTGAGGGTTAATATGC
5C_mPcdh_REV_25	CTTCTCCTTTGCGGCGGGGGTCTTTCCTTTTCCCTTTAGTGAGGGTTAATATGC
5C_mPcdh_REV_26	CTTAAAGTTCACTCTAGGTAATAGTTGCATTCCCTTTAGTGAGGGTTAATATGC
5C_mPcdh_REV_27	CTTCCCCACTCTTGTTACTATTAACAGTTTTCCCTTTAGTGAGGGTTAATATGC
5C_mPcdh_REV_28	CTTGGAAATTTCTTCTCTTAACAGGTGCTTTCCCTTTAGTGAGGGTTAATATGC
5C_mPcdh_REV_29	CTTGTGCTTAAGGGATATTTAATTAATAAATCCCTTTAGTGAGGGTTAATATGC
5C_mPcdh_REV_30	CTTGAAGAGACTGGAAAGAAAGAACTTGGGTCCCTTTAGTGAGGGTTAATATGC
5C_mPcdh_REV_31	CTTCAAGGTGAGTGCCATTTTGATTTCTCCTCCCTTTAGTGAGGGTTAATATGC
5C_mPcdh_REV_32	CTTCTGTCAGTGACCAGCGAGTAAGAATTCTCCCTTTAGTGAGGGTTAATATGC
5C_mPcdh_REV_33	CTTTGCACTTTAGTGAACAAGAATGTATCATCCCTTTAGTGAGGGTTAATATGC

5C_mPcdh_REV_34	CTTTTACGTACAGTTTAGGAGAGAGGTGTGTCCCTTTAGTGAGGGTTAATATGC
5C_mPcdh_REV_35	CTTTGATTGAACAAAGTGCCTTGTTACCAATCCCTTTAGTGAGGGTTAATATGC
5C_mPcdh_REV_36	CTTTCCACCGTGAAAAGAATCTATGAGAAGTCCCTTTAGTGAGGGTTAATATGC
5C_mPcdh_REV_37	CTTCCTTCTTCGTTGTCTGTATAAATCAATTCCCTTTAGTGAGGGTTAATATGC
5C_mPcdh_REV_38	CTTTGAAATGCTGAACTCTGATTTCTTCTTTCCCTTTAGTGAGGGTTAATATGC
5C_mPcdh_REV_39	CTTCCGAAATTCTGCTTTTCAGGTTAATGATCCCTTTAGTGAGGGTTAATATGC
5C_mPcdh_REV_40	CTTAAACAGAATTTACTAGCTTACTAAAGTTCCCTTTAGTGAGGGTTAATATGC
5C_mPcdh_REV_41	CTTACTTAACCTGTATAACACATATATAGGTCCCTTTAGTGAGGGTTAATATGC
5C_mPcdh_REV_42	CTTAGCAAGCATGGCTCTGTATGCCCAGTGTCCCTTTAGTGAGGGTTAATATGC
5C_mPcdh_REV_43	CTTTCTGTTAATGATGAAATCCAAAACTATTCCCTTTAGTGAGGGTTAATATGC
5C_mPcdh_REV_44	CTTACAAGTCAAGATTGAATGATTAAATGATCCCTTTAGTGAGGGTTAATATGC
5C_mPcdh_REV_45	CTTTGTCCAGAACCAGCTCCGGGTATATCTTCCCTTTAGTGAGGGTTAATATGC
5C_mPcdh_REV_46	CTTAAGGCTGGAAAGCAAGTTCAAGGCTGATCCCTTTAGTGAGGGTTAATATGC
5C_mPcdh_REV_47	CTTCACACGTCCGTTCTATGCTACTGTAATTCCCTTTAGTGAGGGTTAATATGC
5C_mPcdh_REV_48	CTTTTAAATTATGGTGGGGATGATGACCCATCCCTTTAGTGAGGGTTAATATGC
5C_mPcdh_REV_49	CTTCTATGTATTTACTGTAGGGGCCTTGTTTCCCTTTAGTGAGGGTTAATATGC
5C_mPcdh_REV_50	CTTTTCTGGAAACAGCCTATACAGGGCAAGTCCCTTTAGTGAGGGTTAATATGC
5C_mPcdh_REV_51	CTTCCATATTACACTTCAGCTGTAACAGGTTCCCTTTAGTGAGGGTTAATATGC
5C_mPcdh_REV_52	CTTGGGTTTGGGCTCTCCTCCAACGGTTAGTCCCTTTAGTGAGGGTTAATATGC
5C_mPcdh_REV_53	CTTCATTTCATCCTCCATAAGGATGTTAAATCCCTTTAGTGAGGGTTAATATGC
5C_mPcdh_REV_54	CTTTTGCTTTATGCCCAGAACCCCTTCTTCTCCCTTTAGTGAGGGTTAATATGC

5C_mPcdh_REV_55	CTTGCACCCCATCTAGACCCACAAAGTGTCTCCCTTTAGTGAGGGTTAATATGC
5C_mPcdh_REV_56	CTTAAATGTTTTTCACTACATATAAATTGTTCCCTTTAGTGAGGGTTAATATGC
5C_mPcdh_REV_57	CTTGTAACCCTGGAGGGAATTCACACCCTGTCCCTTTAGTGAGGGTTAATATGC
5C_mPcdh_REV_58	CTTTCCTTTGATCACCTCCCCCACCCGGGTTCCCTTTAGTGAGGGTTAATATGC
5C_mPcdh_REV_59	CTTTGCTGGGGTTTGGAAATGATAGCTGAATCCCTTTAGTGAGGGTTAATATGC
5C_mPcdh_REV_60	CTTCCTTTAATTTGATTGTTGTTTCGTTTATCCCTTTAGTGAGGGTTAATATGC
5C_mPcdh_REV_61	CTTTTCCCGATCCGCCAGGCGGGGTCACACTCCCTTTAGTGAGGGTTAATATGC
5C_mPcdh_REV_62	CTTGAGAGCCAGCAGTACCATGACAAAGATTCCCTTTAGTGAGGGTTAATATGC
5C_mPcdh_REV_63	CTTCCGTACAAAGGTCTCGCGGAGGCTGGTTCCCTTTAGTGAGGGTTAATATGC
5C_mPcdh_REV_64	CTTGGATCTCAGCTGCCTTGAAACTGGTCTTCCCTTTAGTGAGGGTTAATATGC
5C_mPcdh_REV_65	CTTTCCAGTAGTGGGATGAAGGCTAAAGAGTCCCTTTAGTGAGGGTTAATATGC
5C_mPcdh_REV_66	CTTTTTTGCTTGAACCAACAAAAATGTGTCTCCCTTTAGTGAGGGTTAATATGC
5C_mPcdh_REV_67	CTTATGCATGCTGTTTGACTATAGAAATAATCCCTTTAGTGAGGGTTAATATGC
5C_mPcdh_REV_68	CTTAGTGGGGAAACTGCACTGGTCCTGCCCTCCCTTTAGTGAGGGTTAATATGC
5C_mPcdh_REV_69	CTTAAAAACTTTGTGAGAACAAGGGGTATTTCCCTTTAGTGAGGGTTAATATGC
5C_mPcdh_REV_70	CTTATTAACAAGAACACTTCCTCTTTAGTGTCCCTTTAGTGAGGGTTAATATGC
5C_mPcdh_REV_71	CTTGCAAGTTCAGTTAGCAAGTACCCATGCTCCCTTTAGTGAGGGTTAATATGC
5C_mPcdh_REV_72	CTTAGCTGCCAGTGTAGTTGGAAATCTGGGTCCCTTTAGTGAGGGTTAATATGC
5C_mPcdh_REV_73	CTTTATTTCATGGTTATAGCTCAATTCTAATCCCTTTAGTGAGGGTTAATATGC
5C_mPcdh_REV_74	CTTTGGTGTGCATAAGGTTTGGTAAGGGGATCCCTTTAGTGAGGGTTAATATGC
5C_mPcdh_REV_75	CTTTTCTTCGTCAGTAGATATTAAACTGCTTCCCTTTAGTGAGGGTTAATATGC

5C_mPcdh_REV_76	CTTCTTAAATGATAATAACTGATGTGACTATCCCTTTAGTGAGGGTTAATATGC
5C_mPcdh_REV_77	CTTGAACACATTTTTACACTTTAAGTAAATTCCCTTTAGTGAGGGTTAATATGC
5C_mPcdh_REV_78	CTTAGAGCTGTTTTGTTGTCCTTAATTAGATCCCTTTAGTGAGGGTTAATATGC
5C_mPcdh_REV_79	CTTTGAAAGATACCAAGGGGGGGGGACACCTCCCTTTAGTGAGGGTTAATATGC
5C_mPcdh_REV_80	CTTAGAACAGACATTCTGGTGGCGACACCCTCCCTTTAGTGAGGGTTAATATGC
5C_mPcdh_FOR_1	CGGTAATACGACTCACTATAGCCATGCTTCCTTCATTATGATGGTGATGAAAG
5C_mPcdh_FOR_2	CGGTAATACGACTCACTATAGCCCAGTGATAATTAAAAAGGAAGGGAAGGAAG
5C_mPcdh_FOR_3	CGGTAATACGACTCACTATAGCCTCCACGGCTCCCAGGTTTGCAAAGGGAAAG
5C_mPcdh_FOR_4	CGGTAATACGACTCACTATAGCCAAAATTTCTGCCGAGAAATCTGCTGATAAG
5C_mPcdh_FOR_5	CGGTAATACGACTCACTATAGCCCATTTAATGGGCCTCCTTATGGAGTACAAG
5C_mPcdh_FOR_6	CGGTAATACGACTCACTATAGCCTTCCCTCCCCAGATAATAGAGCACCCTAAG
5C_mPcdh_FOR_7	CGGTAATACGACTCACTATAGCCGGGACCAGGAGAACGGCCAGGTGCCAAAAG
5C_mPcdh_FOR_8	CGGTAATACGACTCACTATAGCCCTAGCTATTGAAATATGAACTAGTTAGAAG
5C_mPcdh_FOR_9	CGGTAATACGACTCACTATAGCCAGCAGGTTATTGCTATTTTTGACTGAAAAG
5C_mPcdh_FOR_10	CGGTAATACGACTCACTATAGCCGTCCATCCAATTCATTTGGTGTCCTGTAAG
5C_mPcdh_FOR_11	CGGTAATACGACTCACTATAGCCAGAACTCACACAGACTAGAAATGTTTCAAG
5C_mPcdh_FOR_12	CGGTAATACGACTCACTATAGCCAGAGACTTTCTCAGACATATCTCTATCAAG
5C_mPcdh_FOR_13	CGGTAATACGACTCACTATAGCCCAAATACAAGGAAATGGTTGGACAGAGAAG
5C_mPcdh_FOR_14	CGGTAATACGACTCACTATAGCCTGGCCATGAATGTGATGACAATTAGTGAAG
5C_mPcdh_FOR_15	CGGTAATACGACTCACTATAGCCATTTTCCTGCCATATGTATAACATACAAAG
5C_mPcdh_FOR_16	CGGTAATACGACTCACTATAGCCGTCTGATAATCTTTGCCTTTTGGAATTAAG

5C_mPcdh_FOR_17	CGGTAATACGACTCACTATAGCCAGTCGAAACCCCCCAGCTGAGTGCTGGAAG
5C_mPcdh_FOR_18	CGGTAATACGACTCACTATAGCCATGAGTGTTACTCAATCATAGAAAGATAAG
5C_mPcdh_FOR_19	CGGTAATACGACTCACTATAGCCTCAAACCTAGGTCAATGTCTAAAGAAAAAG
5C_mPcdh_FOR_20	CGGTAATACGACTCACTATAGCCCCTTGAAGAAAAGGAAAATGCTTCTCCAAG
5C_mPcdh_FOR_21	CGGTAATACGACTCACTATAGCCCCAGTTTTATTTATCGACAAGCAAATAAAG
5C_mPcdh_FOR_22	CGGTAATACGACTCACTATAGCCAATGTCTGACTCTGTTGTACTCAGGTCAAG
5C_mPcdh_FOR_23	CGGTAATACGACTCACTATAGCCAGCTAGGTATCTTGGTAACAAAGTTTCAAG
5C_mPcdh_FOR_24	CGGTAATACGACTCACTATAGCCATATCTCTGTGGCCACTCTGGGGAAGGAAG
5C_mPcdh_FOR_25	CGGTAATACGACTCACTATAGCCATTGTGACAAAACCATCCCCACCGGCAAAG
5C_mPcdh_FOR_26	CGGTAATACGACTCACTATAGCCTTAACAAGTAGATAGATTATCCTTTTGAAG
5C_mPcdh_FOR_27	CGGTAATACGACTCACTATAGCCCCTTAGAACCCAAGTTCAGACTCCCACAAG
5C_mPcdh_FOR_28	CGGTAATACGACTCACTATAGCCAAATGTAATCCCACAGATGGCGTGACGAAG
5C_mPcdh_FOR_29	CGGTAATACGACTCACTATAGCCTAAACTGAGAAGTTTCCAAACACATTCAAG
5C_mPcdh_FOR_30	CGGTAATACGACTCACTATAGCCAAAGAGTGCCCCAATCCCTCCTGGAGAAAG
5C_mPcdh_FOR_31	CGGTAATACGACTCACTATAGCCAAATTGCCTTTCTCATAAGGTTGCTGCAAG
5C_mPcdh_FOR_32	CGGTAATACGACTCACTATAGCCAAGCAAAATTAAATCCTAAAGCCGGTAAAG
5C_mPcdh_FOR_33	CGGTAATACGACTCACTATAGCCATCAATGACAATGCCCCGGTGTTTTCAAAG
5C_mPcdh_FOR_34	CGGTAATACGACTCACTATAGCCCTGGTAAAAATTCCTGCACCATTCTAGAAG
5C_mPcdh_FOR_35	CGGTAATACGACTCACTATAGCCCTTTGATAAATCCATTGGATTACTTTTAAG
5C_mPcdh_FOR_36	CGGTAATACGACTCACTATAGCCAACAACACTCGTAAACATAGTAAGCACAAG
5C_mPcdh_FOR_37	CGGTAATACGACTCACTATAGCCTGGATGGATGTCATGATAAATTAATCCAAG

5C_mPcdh_FOR_38	CGGTAATACGACTCACTATAGCCCGTTATTCAAAGACAAATGGAATCTTAAAG
5C_mPcdh_FOR_39	CGGTAATACGACTCACTATAGCCTTTTGAGATGCAGTCTTTATTTAGCCTAAG
5C_mPcdh_FOR_40	CGGTAATACGACTCACTATAGCCTGCTGGAATTCCATACTTGTGTCATAAAAG
5C_mPcdh_FOR_41	CGGTAATACGACTCACTATAGCCGATGCATTATTTGGTACCTTTTGGACTAAG
5C_mPcdh_FOR_42	CGGTAATACGACTCACTATAGCCCCACCTACTGTCAAATGGAGGCACATAAAG
5C_mPcdh_FOR_43	CGGTAATACGACTCACTATAGCCGGTTTTATTTCTCATTATAGAAAACTGAAG
5C_mPcdh_FOR_44	CGGTAATACGACTCACTATAGCCAGGTCCAGGTAACTGTCTTGTTGCTTCAAG
5C_mPcdh_FOR_45	CGGTAATACGACTCACTATAGCCGGATGACCTTCCATTTATTCTGAAACCAAG
5C_mPcdh_FOR_46	CGGTAATACGACTCACTATAGCCCAAGACATATTAGATACGGACAAAATTAAG
5C_mPcdh_FOR_47	CGGTAATACGACTCACTATAGCCAATATGGGGTAAGAGGTGCTTTCATATAAG
5C_mPcdh_FOR_48	CGGTAATACGACTCACTATAGCCGTTGGCCTTGTAGCCACACAAAAGATGAAG
5C_mPcdh_FOR_49	CGGTAATACGACTCACTATAGCCTAAAAGGTTTTCTTATCGATGGTTTTCAAG
5C_mPcdh_FOR_50	CGGTAATACGACTCACTATAGCCAAGGTGTATACTACCTTGCATGGACCTAAG
5C_mPcdh_FOR_51	CGGTAATACGACTCACTATAGCCTCTGAGGCCAGAAAGCAATTTCTATACAAG
5C_mPcdh_FOR_52	CGGTAATACGACTCACTATAGCCTCTCCCTTCAGCCCAGATCCTGTAGTGAAG
5C_mPcdh_FOR_53	CGGTAATACGACTCACTATAGCCTTGGAAGTCTCATTCCTTAATTTCAAAAAG
5C_mPcdh_FOR_54	CGGTAATACGACTCACTATAGCCAAAGTAATATGTCAGGCTACCAAAGACAAG
5C_mPcdh_FOR_55	CGGTAATACGACTCACTATAGCCACACCTCTCTCACCAGCTCTATCCAGGAAG
5C_mPcdh_FOR_56	CGGTAATACGACTCACTATAGCCCCACTGAGTGAAAGAAGGGAGGTGCAGAAG
5C_mPcdh_FOR_57	CGGTAATACGACTCACTATAGCCCTATTTGTAAATTGCTTTATTGTGTGAAAG
5C_mPcdh_FOR_58	CGGTAATACGACTCACTATAGCCGTCATTTTGTGGGCATTGACGGGGTACAAG

5C_mPcdh_FOR_59	CGGTAATACGACTCACTATAGCCTAGGCAAAGACCTGCCTTTCAAGATCCAAG
5C_mPcdh_FOR_60	CGGTAATACGACTCACTATAGCCTGCACAGTTGGGATTCTCAGGGTCCAAAAG
5C_mPcdh_FOR_61	CGGTAATACGACTCACTATAGCCTTTCAGTTCTGTTATTTGTAAAAGTGCAAG
5C_mPcdh_FOR_62	CGGTAATACGACTCACTATAGCCATTGAATCTTCTGCCAGCAATTACTATAAG
5C_mPcdh_FOR_63	CGGTAATACGACTCACTATAGCCGAGTGTTTGTGGGCGTAGACGGAGTGCAAG
5C_mPcdh_FOR_64	CGGTAATACGACTCACTATAGCCGATTACCACTAGTACTTCTTTGTCCAAAAG
5C_mPcdh_FOR_65	CGGTAATACGACTCACTATAGCCTGGGCAGGGCTTTAGAAGGTTCTGACAAAG
5C_mPcdh_FOR_66	CGGTAATACGACTCACTATAGCCGGGGAGGCACAAACTCCACAAACTCTTAAG
5C_mPcdh_FOR_67	CGGTAATACGACTCACTATAGCCCTGTCCATCACAGTGCCCATCCTTATAAAG
5C_mPcdh_FOR_68	CGGTAATACGACTCACTATAGCCCCCTCCCTGTACTGACTTCTCTATAAAAAG
5C_mPcdh_FOR_69	CGGTAATACGACTCACTATAGCCCAGAGAGGATTGCTTTGGGCTAGCAAAAAG
5C_mPcdh_FOR_70	CGGTAATACGACTCACTATAGCCCTTATCTGTAAGGTTTACTTCTGTAATAAG
5C_mPcdh_FOR_71	CGGTAATACGACTCACTATAGCCGTTAGTTACCCTTTCGTGCCCACTAAAAAG
5C_mPcdh_FOR_72	CGGTAATACGACTCACTATAGCCCATCACCAACATCTAAGCTCAGTGGCAAAG
5C_mPcdh_FOR_73	CGGTAATACGACTCACTATAGCCTTAACAGTGGGTACATCCACAGTCCCAAAG
5C_mPcdh_FOR_74	CGGTAATACGACTCACTATAGCCTAACATAAAATGGGGACATGGTGGTATAAG
5C_mPcdh_FOR_75	CGGTAATACGACTCACTATAGCCTTCATTCTTCCTACTTCAGATACTCAAAAG
5C_mPcdh_FOR_76	CGGTAATACGACTCACTATAGCCTAAAGCAAACATCCCTAAACAACTAACAAG
5C_mPcdh_FOR_77	CGGTAATACGACTCACTATAGCCACTCTGTCACTTCCAGACAGCTATATAAAG
5C_mPcdh_FOR_78	CGGTAATACGACTCACTATAGCCGACTGGGGCTGCAACCACTCAAAGACAAAG
5C_mPcdh_FOR_79	CGGTAATACGACTCACTATAGCCTAGACTAGATTTCCATACAATCTCTCAAAG

5C_mPcdh_FOR_80	CGGTAATACGACTCACTATAGCCCAAAGGTTTTAGACCAAATCTTGTACCAAG
5C-T7-F1	AATGATACGGCGACCACCGAGATCTACACTCTTTCCCTACACGACGCTCTTCCGATCTCGGTAATACGACTCACTATAGCC
5C-T7-F2-A	AATGATACGGCGACCACCGAGATCTACACTCTTTCCCTACACGACGCTCTTCCGATCTACGGTAATACGACTCACTATAGCC
5C-T7-F3-TG	AATGATACGGCGACCACCGAGATCTACACTCTTTCCCTACACGACGCTCTTCCGATCTTGCGGTAATACGACTCACTATAGCC
5C-T7-F4-TAA	AATGATACGGCGACCACCGAGATCTACACTCTTTCCCTACACGACGCTCTTCCGATCTTAACGGTAATACGACTCACTATAGCC
5C-T7-F5-CTAG	AATGATACGGCGACCACCGAGATCTACACTCTTTCCCTACACGACGCTCTTCCGATCTCTAGCGGTAATACGACTCACTATAGCC
5C-T3-R	CAAGCAGAAGACGGCATACGAGATTCAAGTGTGACTGGAGTTCAGACGTGTGCTCTTCCGATCTGCATATTAACCCTCACTAAAGGGA
5C-T3-R2-AAGCTA	CAAGCAGAAGACGGCATACGAGATAAGCTAGTGACTGGAGTTCAGACGTGTGCTCTTCCGATCTGCATATTAACCCTCACTAAAGGGA
**Oligonucleotides used in constructing sgRNAs for *Pcdhγ* CBSs *b-e* inversion mice**	
CR-T7-ginvb-e-gRNA1-F	TAATACGACTCACTATAGGGACAGACATAGTCGCTTTGCCGTTTTAGAGCTAGAAATAG
CR-T7-ginvb-e-gRNA2-F	TAATACGACTCACTATAGGGCTAAGAGAGGCCGATACGTTTTAGAGCTAGAAATAG
**Oligonucleotides used in constructing sgRNAs for screening single-cell CRISPR inversion clones**	
OutsideCBS15sgRNA1F	accgACCCAATGACCTCAGGCTGT
OutsideCBS15sgRNA1R	aaacACAGCCTGAGGTCATTGGGT
betweenCBS14-15sgRNA2F	accgGCCTTTCCTAAGGGTCTGTG
betweenCBS14-15sgRNA2R	aaacCACAGACCCTTAGGAAAGGC
betweenCBS13-14sgRNA3F	accgGCACTGCCGAGCCTACACTG
betweenCBS13-14sgRNA3R	aaacCAGTGTAGGCTCGGCAGTGC
OutsideCBS13sgRNA4F	accgTCACTTGTTAGCGGCATCTG
OutsideCBS13sgRNA4R	aaacCAGATGCCGCTAACAAGTGA
outsideHS5-1asgRNA1F	accgCCACACATCCAAGGCTGAC

outsideHS5-1asgRNA1R	aaacGTCAGCCTTGGATGTGTGG
betweenHS5-1absgRNA2F	accgAGAAAGCAATCCATATGGTA
betweenHS5-1absgRNA2R	aaacTACCATATGGATTGCTTTCT
betweenHS5-1absgRNA3F	accgGCTTCCGGTAGGGCGGGGTC
betweenHS5-1absgRNA3R	aaacGACCCCGCCCTACCGGAAGC
outsideHS5-1bsgRNA4F	accgAGATTTGGGGCGTCAGGAAG
outsideHS5-1bsgRNA4R	aaacCTTCCTGACGCCCCAAATCT
**Primers for screening single-cell CRISPR inversion clones of different combinations of CBS sites**	
outCBS15-1F	AGGTTGAATGAATGCGTGACTG
outCBS15-1F2	CTGCCTCTTTATGGGTCTAATGTAC
outCBS15-1R	AGAGCCACCAGTCCACAGATC
outCBS15-1R2	ACGCAGGAGCCGTATCATG
outCBS13-3F2	ATAGCAATGAAATCTTGAAGGAGTG
outCBS13-3R2	GCACAGCCCTGCTCTATTACG
CBS15F1	TGAGACCCGCTAGGAAATGG
CBS15R1	CCCACAACTCCCTTTCAATCAG
CBS14F1	AGTGGAGCACCCTCACATCC
CBS14F2	GCGCTCAGTGTAGAGCTCGTG
CBS14R1	GGATCGGCTGTTTGCTAGGTC
HS51-IF1	CCCTCCACCTCTGGCATTG
HS51-IF2	CGAGTCATGGGACCGAACTG
HS51-IR1	TTTTTGGCTAACAACATAGTGCTTC
HS51-IR2	TTATCAATAGCATTTTCCTCATCTG
HS51-IIF1	GCAAGGAGATCCGTGTCGTC
HS51-IIF2	CGAGTCATGGGACCGAACTG
HS51-IIR1	CGGACTACATTTGCTTTTTCTCG
HS51-IIR2	TGAGTAGAAGCGAGAGATCACTCTG
HS51-IIIF1	CTGCAACAACCCCTGCAATC
HS51-IIIF2	TCGCCCTCTGCTGGTTAAAG
HS51-IIIR1	AGCTGAGGAAGGTGTTGTGG
HS51-IIIR2	GCAGAAACCAGGGGCAAATG
HS51-IVF1	TTCATCCCCGCTTCCTACTG
HS51-IVF2	CCCTCCACCTCTGGCATTG
HS51-IVR1	TGCTTTCTCATTCCCCGTTG

HS51-IVR2	GTGTTGGAAAAAATGCTTGGAG
**Primers for 4C at the *HS5-1* enhancer**	
4C_hHD_HS5-1_F_a	AATGATACGGCGACCACCGAGATCTACACTCTTTCCCTACACGACGCTCTTCCGATCTTAGCCAAAAATATTCCAGGAAG
4C_hHD_HS5-1_F_b	AATGATACGGCGACCACCGAGATCTACACTCTTTCCCTACACGACGCTCTTCCGATCTTGCATAGCCAAAAATATTCCAGGAAG
4C_hHD_HS5-1_F_c	AATGATACGGCGACCACCGAGATCTACACTCTTTCCCTACACGACGCTCTTCCGATCTGCTATAGCCAAAAATATTCCAGGAAG
4C_hHD_HS5-1_F_d	AATGATACGGCGACCACCGAGATCTACACTCTTTCCCTACACGACGCTCTTCCGATCTACGCTAGCCAAAAATATTCCAGGAAG
4C_hHD_HS5-1_F_e	AATGATACGGCGACCACCGAGATCTACACTCTTTCCCTACACGACGCTCTTCCGATCTCGATTAGCCAAAAATATTCCAGGAAG
4C_hHD_HS5-1_F_f	AATGATACGGCGACCACCGAGATCTACACTCTTTCCCTACACGACGCTCTTCCGATCTGATCTAGCCAAAAATATTCCAGGAAG
4C_hHD_HS5-1_R-a	CAAGCAGAAGACGGCATACGAGATTGACATGTGACTGGAGTTCAGACGTGTGCTCTTCCGATCTGTGTTGGAAAAAATGCTTGGAG
4C_hHD_HS5-1_R-b	CAAGCAGAAGACGGCATACGAGATCGTACGGTGACTGGAGTTCAGACGTGTGCTCTTCCGATCTGTGTTGGAAAAAATGCTTGGAG
4C_hHD_HS5-1_R-c	CAAGCAGAAGACGGCATACGAGATACATCGGTGACTGGAGTTCAGACGTGTGCTCTTCCGATCTGTGTTGGAAAAAATGCTTGGAG
4C_hHD_HS5-1_R-d	CAAGCAGAAGACGGCATACGAGATGATCTGGTGACTGGAGTTCAGACGTGTGCTCTTCCGATCTGTGTTGGAAAAAATGCTTGGAG
4C_hHD_HS5-1_R-e	CAAGCAGAAGACGGCATACGAGATTCAAGTGTGACTGGAGTTCAGACGTGTGCTCTTCCGATCTGTGTTGGAAAAAATGCTTGGAG
4C_hHD_HS5-1_R-f	CAAGCAGAAGACGGCATACGAGATCTGATCGTGACTGGAGTTCAGACGTGTGCTCTTCCGATCTGTGTTGGAAAAAATGCTTGGAG
**Primers for 4C at the *β-globin* gene locus**	
4C_hEN_CBS 13-15_F_A1	AATGATACGGCGACCACCGAGATCTACACTCTTTCCCTACACGACGCTCTTCCGATCTTCACGCCCTGAAGCTTGTCTGGAG
4C_hEN_CBS 13-15_F_A2	AATGATACGGCGACCACCGAGATCTACACTCTTTCCCTACACGACGCTCTTCCGATCTATGCTCACGCCCTGAAGCTTGTCTGGAG
4C_hEN_CBS 13-15_F_A3	AATGATACGGCGACCACCGAGATCTACACTCTTTCCCTACACGACGCTCTTCCGATCTATGCGCCCTGAAGCTTGTCTGGAG
4C_hEN_CBS 13-15_F_A4	AATGATACGGCGACCACCGAGATCTACACTCTTTCCCTACACGACGCTCTTCCGATCTTGCAGCCCTGAAGCTTGTCTGGAG
4C_hEN_CBS 13-15_F_A5	AATGATACGGCGACCACCGAGATCTACACTCTTTCCCTACACGACGCTCTTCCGATCTGCTAGCCCTGAAGCTTGTCTGGAG
4C_hEN_CBS 13-15_F_A6	AATGATACGGCGACCACCGAGATCTACACTCTTTCCCTACACGACGCTCTTCCGATCTCAGTGCCCTGAAGCTTGTCTGGAG
4C_hEN_CBS 13-15_R-B1	CAAGCAGAAGACGGCATACGAGATCGTGATGTGACTGGAGTTCAGACGTGTGCTCTTCCGATCTCTCATTTGGGGTGTTATATGC

4C_hEN_CBS 13-15_R-B2	CAAGCAGAAGACGGCATACGAGATATCACGGTGACTGGAGTTCAGACGTGTGCTCTTCCGATCTCTCATTTGGGGTGTTATATGC
4C_hEN_CBS 13-15_R-B3	CAAGCAGAAGACGGCATACGAGATCGATGTGTGACTGGAGTTCAGACGTGTGCTCTTCCGATCTCTCATTTGGGGTGTTATATGC
**Primers used for 4C for *Pcdh*α and *Pcdh*$\alpha\beta$ deletion mice**	
4C_mHiDp_ga 3F	CAAGCAGAAGACGGCATACGAGATCGTGATGTGACTGGAGTTCAGACGTGTGCTCTTCCGATCCATCGTGGGAATCAGAGG
4C_mHiDp_ga 3R1	AATGATACGGCGACCACCGAGATCTACACTCTTTCCCTACACGACGCTCTTCCGATCTTTATGGATTATAATTCTTGAAGC
4C_mHiDp_ga 3R2-GAT	AATGATACGGCGACCACCGAGATCTACACTCTTTCCCTACACGACGCTCTTCCGATCTGATTTATGGATTATAATTCTTGAAGC
4C_mHiDp_ga 3R3-GCG	AATGATACGGCGACCACCGAGATCTACACTCTTTCCCTACACGACGCTCTTCCGATCTGCGTTATGGATTATAATTCTTGAAGC
4C_mHiDp_ga 3R4-CTA	AATGATACGGCGACCACCGAGATCTACACTCTTTCCCTACACGACGCTCTTCCGATCTCTATTATGGATTATAATTCTTGAAGC
4C_mHiDp_ga 3R5-CGC	AATGATACGGCGACCACCGAGATCTACACTCTTTCCCTACACGACGCTCTTCCGATCTCGCTTATGGATTATAATTCTTGAAGC
4C_mHiDp_ga 3R6-TCACA	AATGATACGGCGACCACCGAGATCTACACTCTTTCCCTACACGACGCTCTTCCGATCTTCACATTATGGATTATAATTCTTGAAGC
4C_mHiDp_ga 3R7-TGCAT	AATGATACGGCGACCACCGAGATCTACACTCTTTCCCTACACGACGCTCTTCCGATCTTGCATTTATGGATTATAATTCTTGAAGC
4C_mHiDp_ga 3R8-ATGTG	AATGATACGGCGACCACCGAGATCTACACTCTTTCCCTACACGACGCTCTTCCGATCTATGTGTTATGGATTATAATTCTTGAAGC
4C_mHiDp_ga 3R9-AATGC	AATGATACGGCGACCACCGAGATCTACACTCTTTCCCTACACGACGCTCTTCCGATCTAATGCTTATGGATTATAATTCTTGAAGC
4C_mHiDp_H S7LF	CAAGCAGAAGACGGCATACGAGATCGTGATGTGACTGGAGTTCAGACGTGTGCTCTTCCGATCGCTGTCTGGGAACCCACTC
4C_mHiDp_H S7LR1	AATGATACGGCGACCACCGAGATCTACACTCTTTCCCTACACGACGCTCTTCCGATCTGCTGTGACAGAGGTTCTTTCTAAG
4C_mHiDp_H S7LR2-AGA	AATGATACGGCGACCACCGAGATCTACACTCTTTCCCTACACGACGCTCTTCCGATCTAGAGCTGTGACAGAGGTTCTTTCTAAG
4C_mHiNl_HS 18-20F	CAAGCAGAAGACGGCATACGAGATCGTGATGTGACTGGAGTTCAGACGTGTGCTCTTCCGATCAATCCCAACCTAAGACAGC
4C_mHiNl_HS 18-20R1	AATGATACGGCGACCACCGAGATCTACACTCTTTCCCTACACGACGCTCTTCCGATCTATTCAATCAGGCCTTTTAAGCT
4C_mHiNl_HS 18-20R2-CTG	AATGATACGGCGACCACCGAGATCTACACTCTTTCCCTACACGACGCTCTTCCGATCTCTGATTCAATCAGGCCTTTTAAGCT
4C_mHiDp_H S17F	AATGATACGGCGACCACCGAGATCTACACTCTTTCCCTACACGACGCTCTTCCGATCTGTAAGGTTTACTTCTGTAATAAGC
4C_mHiDp_H S17F2-TCT	AATGATACGGCGACCACCGAGATCTACACTCTTTCCCTACACGACGCTCTTCCGATCTTCTGTAAGGTTTACTTCTGTAATAAGC

4C_mHiDp_HS17F3-TAG	AATGATACGGCGACCACCGAGATCTACACTCTTTCCCTACACGACGCTCTTCCGATCTTAGGTAAGGTTTACTTCTGTAATAAGC
4C_mHiDp_HS17F4-ATC	AATGATACGGCGACCACCGAGATCTACACTCTTTCCCTACACGACGCTCTTCCGATCTATCGTAAGGTTTACTTCTGTAATAAGC
4C_mHiDp_HS17F5-AGA	AATGATACGGCGACCACCGAGATCTACACTCTTTCCCTACACGACGCTCTTCCGATCTAGAGTAAGGTTTACTTCTGTAATAAGC
4C_mHiDp_HS17F6-GCA	AATGATACGGCGACCACCGAGATCTACACTCTTTCCCTACACGACGCTCTTCCGATCTGCAGTAAGGTTTACTTCTGTAATAAGC
4C_mHiDp_HS17F7-GAC	AATGATACGGCGACCACCGAGATCTACACTCTTTCCCTACACGACGCTCTTCCGATCTGACGTAAGGTTTACTTCTGTAATAAGC
4C_mHiDp_HS17F8-CGT	AATGATACGGCGACCACCGAGATCTACACTCTTTCCCTACACGACGCTCTTCCGATCTCGTGTAAGGTTTACTTCTGTAATAAGC
4C_mHiDp_HS17R	CAAGCAGAAGACGGCATACGAGATCGTGATGTGACTGGAGTTCAGACGTGTGCTCTTCCGATCGCCCTGAGCATTCAGAGATC
4C_mHiDp_HS5-1F	AATGATACGGCGACCACCGAGATCTACACTCTTTCCCTACACGACGCTCTTCCGATCTCCCTCAATCATTAAATTTCTATTAAAGC
4C_mHiDp_HS5-1R	CAAGCAGAAGACGGCATACGAGATCGTGATGTGACTGGAGTTCAGACGTGTGCTCTTCCGATCGACTTCCTTTCATTGTCCCATTC
4C_mHD_HS5-1F3-AGA	AATGATACGGCGACCACCGAGATCTACACTCTTTCCCTACACGACGCTCTTCCGATCTAGACCCTCAATCATTAAATTTCTATTAAAGC
4C_mHD_HS5-1F4-TAG	AATGATACGGCGACCACCGAGATCTACACTCTTTCCCTACACGACGCTCTTCCGATCTTAGCCCTCAATCATTAAATTTCTATTAAAGC
4C_mHD_HS5-1F5-GAT	AATGATACGGCGACCACCGAGATCTACACTCTTTCCCTACACGACGCTCTTCCGATCTGATCCCTCAATCATTAAATTTCTATTAAAGC
4C_mHD_HS5-1F6-CTA	AATGATACGGCGACCACCGAGATCTACACTCTTTCCCTACACGACGCTCTTCCGATCTCTACCCTCAATCATTAAATTTCTATTAAAGC
**Primers for RNA-seq**	
RNAseq-index-universal	AATGATACGGCGACCACCGAGATCTACACTCTTTCCCTACACGACGCTCTTCCGATCT
RNAseq-Index-P3	CAAGCAGAAGACGGCATACGAGATCGAGTAGTGACTGGAGTTCAGACGTGTGCTCTTCCGATCT
RNAseq-Index-P4	CAAGCAGAAGACGGCATACGAGATTCTCCGGTGACTGGAGTTCAGACGTGTGCTCTTCCGATCT
RNAseq-Index-P5	CAAGCAGAAGACGGCATACGAGATAATGAGGTGACTGGAGTTCAGACGTGTGCTCTTCCGATCT
RNAseq-Index-P6	CAAGCAGAAGACGGCATACGAGATGGAATCGTGACTGGAGTTCAGACGTGTGCTCTTCCGATCT
RNAseq-Index-P7	CAAGCAGAAGACGGCATACGAGATTTCTGAGTGACTGGAGTTCAGACGTGTGCTCTTCCGATCT
RNAseq-Index-P8	CAAGCAGAAGACGGCATACGAGATACGAATGTGACTGGAGTTCAGACGTGTGCTCTTCCGATCT

RNAseq-Index-P9	CAAGCAGAAGACGGCATACGAGATAGCTTCGTGACTGGAGTTCAGACGTGTGCTCTTCCGATCT
RNAseq-Index-P10	CAAGCAGAAGACGGCATACGAGATGCGCATGTGACTGGAGTTCAGACGTGTGCTCTTCCGATCT
RNAseq-Index-P11	CAAGCAGAAGACGGCATACGAGATCATAGCGTGACTGGAGTTCAGACGTGTGCTCTTCCGATCT
RNAseq-Index-P12	CAAGCAGAAGACGGCATACGAGATTTCGCGGTGACTGGAGTTCAGACGTGTGCTCTTCCGATCT
RNAseq-Index-P13	CAAGCAGAAGACGGCATACGAGATTCAAGTGTGACTGGAGTTCAGACGTGTGCTCTTCCGATCT
RNAseq-Index-P14	CAAGCAGAAGACGGCATACGAGATCTATCGGTGACTGGAGTTCAGACGTGTGCTCTTCCGATCT
RNAseq-Index-P15	CAAGCAGAAGACGGCATACGAGATACATCTGTGACTGGAGTTCAGACGTGTGCTCTTCCGATCT
RNAseq-Index-P16	CAAGCAGAAGACGGCATACGAGATGTTGACGTGACTGGAGTTCAGACGTGTGCTCTTCCGATCT
